# *N*-Cyclo­amino substituent effects on the packing architecture of *ortho*-sul­fan­il­amide mol­ecular crystals and their *in silico* carbonic anhydrase II and IX inhibitory activities

**DOI:** 10.1107/S2053229622010130

**Published:** 2022-11-08

**Authors:** Sherif O. Kolade, Josephat U. Izunobi, Allen T. Gordon, Eric C. Hosten, Idris A. Olasupo, Adeniyi S. Ogunlaja, Olayinka T. Asekun, Oluwole B. Familoni

**Affiliations:** a Department of Chemistry, University of Lagos, Akoka-Yaba, Lagos, Nigeria; b Department of Chemistry, Nelson Mandela University, Port Elizabeth, 6031, South Africa; J-PARC Center, Japan Atomic Energy Agency, Japan

**Keywords:** sul­fan­il­amide, nitro­sul­fonamide, cyclo­amino, SC-XRD, DFT, crystal structure, packing architecture, inhibitory activity

## Abstract

In the search for new ‘sulfa drugs’ with therapeutic properties, *o*-nitro­sul­fonamides and *N*-cyclo­amino-*o*-sul­fan­il­amides were synthesized and characterized using techniques including ^1^H NMR, ^13^C NMR and FT–IR spectroscopy, and single-crystal X-ray diffraction (SC-XRD). The calculated density functional theory (DFT)-optimized geometry of the mol­ecules showed similar conformations to those obtained by SC-XRD. Mol­ecular docking of *N*-piperidinyl-*o*-sul­fan­il­amide and *N*-indolinyl-*o*-sul­fan­il­amide supports the notion that *o*-sul­fan­il­amides are able to bind to human carbonic anhydrase II and IX inhibitors (hCA II and IX; PDB entries 4iwz and 5fl4). Hirshfeld surface analyses and DFT studies of three *o*-nitro­sul­fonamides {1-[(2-nitro­phen­yl)sul­fon­yl]pyrrolidine, C_10_H_12_N_2_O_4_S, **1**, 1-[(2-nitro­phen­yl)sul­fon­yl]piperidine, C_11_H_14_N_2_O_4_S, **2**, and 1-[(2-nitro­phen­yl)sul­fon­yl]-2,3-di­hydro-1*H*-indole, C_14_H_12_N_2_O_4_S, **3**} and three *N*-cyclo­amino-*o*-sul­fan­il­amides [2-(pyrrolidine-1-sul­fon­yl)aniline, C_10_H_14_N_2_O_2_S, **4**, 2-(piperidine-1-sul­fon­yl)aniline, C_11_H_16_N_2_O_2_S, **5**, and 2-(2,3-di­hydro-1*H*-indole-1-sul­fon­yl)aniline, C_14_H_14_N_2_O_2_S, **6**] suggested that forces such as hydro­gen bonding and π–π inter­actions hold mol­ecules together and further showed that charge transfer could promote bioactivity and the ability to form biological inter­actions at the piperidinyl and phenyl moieties.

## Abstract 2

Three *o*-nitro­sul­fonamides and three *N*-cyclo­amino-*o*-sul­fan­il­amides have been successfully synthesized and characterized, and the intermolecular interactions analysed, as well as being tested *in silico* for carbonic anhydrase II (4iwz) and IX (5fl4) inhibitory activities. The results obtained from crystal packing and DFT analysis suggest that the mol­ecules are held together by forces such as hydro­gen bonding and π–π inter­actions.

## Introduction

**Figure 1:**
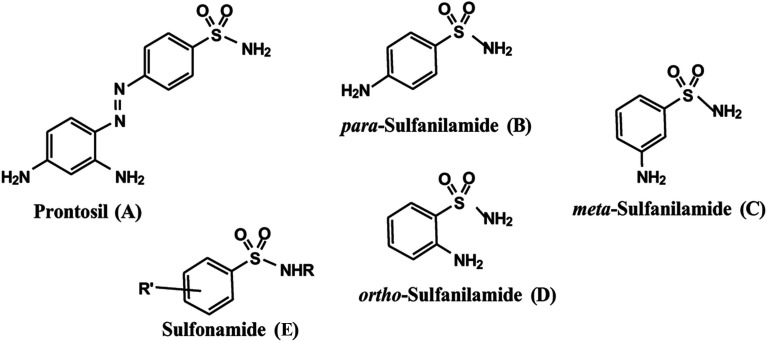
Sulfanilamides and some prodrugs.

Sulfanilamide (4-amino­benzene­sul­fonamide) is aptly des­cribed as the antecedent of the group of therapeutics known as ‘sulfa drugs’, which ushered in the modern era of anti­bacterial chemotherapy (Ajani *et al.*, 2012 ▸). Although it had been a com­ponent of a staple azo dye in the colour industry since the beginning of the 20th century, it did not gain prominence in medicine until the 1930s when Gerhard Domagk and co-workers patented Prontosil, **A** (Fig. 1 ▸), a sul­fan­il­amide pro­drug, which not only revolutionized the treatment of bacterial infections, but chemotherapy as a whole, and led to the development of other drugs for non-infectious diseases.

It has been established that the bacteriostatic properties of sul­fan­il­amides (Fig. 1 ▸) are predicated based on two major motifs: the aryl amine (–NH_2_) and sul­fonamide (–SO_2_NH*R*) groups (Lesch, 2007 ▸). A free or hydrolysable substituted amino (–NH*R*′) moiety that is *para* to the sul­fonamido group has been reported to be crucial for anti­bacterial activity, whereas modification of the position to the *ortho* and/or *meta* position results in non-anti­bacterial activities (Ajani *et al.*, 2012 ▸). The derivatization of the sul­fonamido group with heterocycles has also produced more potent anti­biotics (Ajani *et al.*, 2012 ▸; Lesch, 2007 ▸). In addition, it has been long reported that no correlations exist between the toxicities and therapeutic efficiencies, as well as toxicities and solubilities, of the three isomers of sul­fan­il­amide, as evidenced by the finding that even though *meta*-sul­fan­il­amide **C** was the least toxic of the three, only *para*-sul­fan­il­amide **B** possessed bacteriostatic activity (Laug & Morris, 1939 ▸). Notably, the inhibitions of the *Helicobacter pylori* α-class carbonic anhydrase (hpCA) (Nishimori *et al.*, 2006 ▸) and tumour-associated transmembrane carbonic anhydrase IX (CA IX) (Vullo *et al.*, 2003 ▸) isozymes have been observed with *ortho*-sul­fan­il­amide **D** (orthanilam­ide). Sulfonamides **E** are derivatives of sul­fan­il­amide and remain an important class of drugs, with anti­bacterial and non-anti­bacterial potencies, such as diuretic, anti­microbial, anti-epileptic, anti­leprotic, anti­malarial, hypoglycemic, anti­retro­viral, anti­thyroid and anti-inflammatory activities (Gul *et al.*, 2016 ▸; Henry, 1943 ▸; Casini *et al.*, 2002 ▸; Mohan *et al.*, 2006 ▸; Alex & Storer, 2010 ▸).

They also inhibit carbonic anhydrase (Gul *et al.*, 2016 ▸; Ghorab *et al.*, 2014 ▸; Nocentini *et al.*, 2016 ▸) and have been reported to show *in vivo* and/or *in vitro* anti­tumour activities (Boyland, 1946 ▸). Many of these sul­fonamide-based (sulfa) drugs, reported to be in clinical trials, are devoid of the side effects plaguing most of the current pharmacological agents (Casini *et al.*, 2002 ▸; Owa *et al.*, 2002 ▸; Lavanya, 2017 ▸; Andreucci *et al.*, 2019 ▸).

The identification of pharmacologically active moieties in model mol­ecules and lead candidates of physiological significance from a vast array of substances, with the potential of further optimization, is a crucial facet of rational drug design and discovery (Voronin *et al.*, 2020 ▸). The process of optimization, it must be noted, typically involves structure–activity relationship studies that facilitate the selection of mol­ecules with optimal receptor affinities (Bloom & Laubach, 1962 ▸; Kalgutkar *et al.*, 2010 ▸; Sly & Hu, 1995 ▸; Lehtonen *et al.*, 2004 ▸; Żołnowska *et al.*, 2014 ▸; Thiry *et al.*, 2008 ▸; Angeli *et al.*, 2020 ▸; Güzel-Akdemir *et al.*, 2015 ▸; Rutkauskas *et al.*, 2014 ▸; Congiu *et al.*, 2014 ▸; Temperini *et al.*, 2008*a* ▸,*b* ▸; Chiche *et al.*, 2010 ▸; Türeci *et al.*, 1998 ▸; PDB, http://www.rcsb.org/pdb; Berman *et al.*, 2000 ▸). In continuation of the design of potential ‘sulfa drugs’, we report the synthesis, structural and theoretical studies, and docking application of the *o*-nitro­sul­fonamides 1-[(2-nitro­phen­yl)sul­fon­yl]pyrrolidine, **1**, 1-[(2-nitro­phen­yl)sul­fon­yl]pi­peri­dine, **2**, and 1-[(2-nitro­phen­yl)sul­fon­yl]-2,3-di­hydro-1*H*-indole, **3**, and the *N*-cyclo­amino-*o*-sul­fan­il­amides 2-(pyrrolidine-1-sul­fon­yl)aniline, **4**, 2-(piperidine-1-sul­fon­yl)aniline, **5**, and 2-(2,3-di­hydro-1*H*-indole-1-sul­fon­yl)aniline, **6**. The crystal structures, density functional theory (DFT) studies, Hirshfeld surface analysis, mol­ecular electrostatic potential and electronic properties of the title sul­fonamides and sul­fan­il­amides (**1**–**6**) have been discussed. Mol­ecular docking experiments with carbonic anhydrase II (PDB entry 4iwz) and IX (5fl4) active sites were conducted in order to predict their binding inter­actions with **1**–**6** (Scheme 1). 
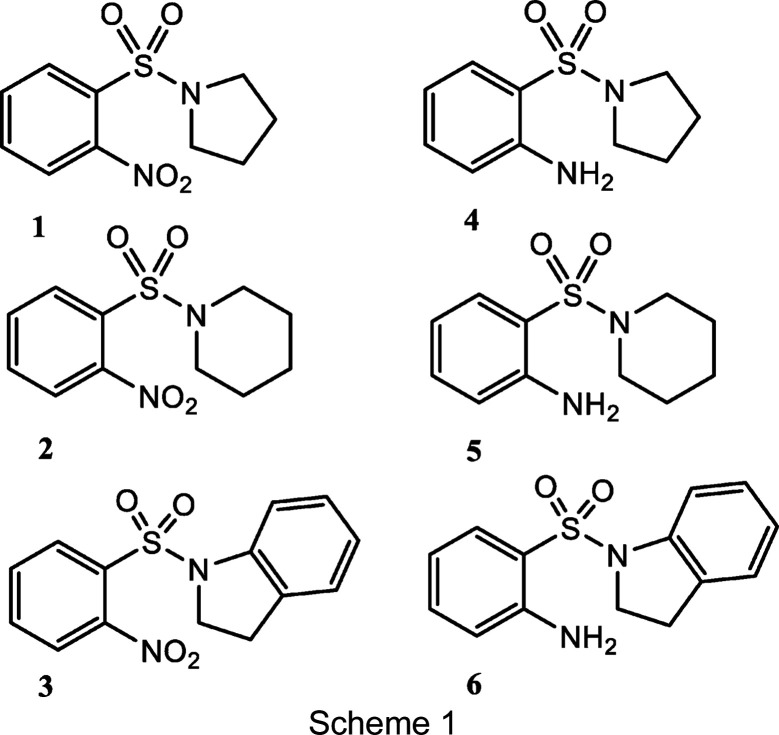


## Experimental

### Instruments and measurements

All reagents were purchased from Millipore Sigma (Ger­many and South Africa) and were used without purification. The melting points were determined on an Electrothermal digital melting-point apparatus and are uncorrected. Reactions were monitored by thin-layer chromatography (TLC) on Merck silica gel 60 F_254_ precoated plates using a di­chloro­methane/*n*-hexane (2 or 1.4:1 *v*/*v*) solvent system visualized under a UV lamp (254 nm). Column chromatography was performed with silica gel (70–230 mesh ASTM) and mobile phases were as indicated. Sample crystallization was achieved by the slow evaporation of the indicated solvent systems at ambient tem­per­ature. IR spectra were obtained using a Bruker Tensor 27 platinum ATR–FT–IR spectrometer. The ATR–FT–IR spectra were acquired in a single mode with a resolution of 4 cm^−1^ over 32 scans, in the region 4000–650 cm^−1^. ^1^H and ^13^C NMR spectra were recorded, in CDCl_3_, on a Bruker 400 MHz spectrometer. Chemical shift (δ) values were measured in parts per million (ppm) downfield from tetra­methyl­silane (TMS) and coupling constants (*J*) are reported in hertz (Hz). Theoretical studies were performed for the com­pounds and, in each case, their SC-XRD structures were used for optimization and global reactivity descriptor (GRD) calculations.

### Synthesis and crystallization

#### Synthesis of *N*-cyclo­amino-*o*-nitro­benzene­sul­fon­amides 1–3

*o*-Nitro­benzene­sul­fonyl chloride (1.00 mmol) was added slowly to a stirring dried toluene solution (30 ml) of the cyclo­amine (2.20 mmol) at ambient tem­per­ature and stirred for 12 h, monitored by TLC. The reaction mixture was then diluted with di­chloro­methane (30 ml) and washed with distilled water (3 × 10 ml). The organic layer was separated, dried over anhydrous sodium sulfate, filtered and concentrated to an oil, which was purified by column chromatography on silica gel (di­chloro­methane/*n*-hexane, 2:1 *v*/*v*). Crystals were obtained by the slow solvent evaporation of the requisite eluates at ambient tem­per­ature, except for **5**, which was recrystallized from di­chloro­methane, slowly evaporated and filtered to give single crystals.

*2.2.1.1. N-Pyrrolidinyl-o-nitro­benzene­sul­fonamide, **1**. o*-Nitro­benzene­sul­fonyl chloride (3.00 g, 13.54 mmol) and pyrrolidine (2.12 g, 2.45 ml, 29.81 mmol). Yellow crystals (2.95 g, 85%); *R*_F_ = 0.44 (CH_2_Cl_2_/*n*-hexane, 2:1 *v*/*v*); m.p. 81.7–81.9 °C. IR (Bruker, ATR, ν, cm^−1^): 3080 (aryl C—H str.), 2968 (*sp* ^3^-C—H str.), 1597 (aryl C=C str.), 1543 (asym C—NO_2_ str.), 1344 (sym C—NO_2_ str.), 1342 (asym SO_2_—N str.), 1163 (sym SO_2_—N str.), 1078 (C—N str.). ^1^H NMR (Bruker, 400 MHz, CDCl_3_, δ_H_, ppm): 7.94 (1H, *d*, *J* = 8 Hz, Ar**H**), 7.62 (2H, *t*, *J* = 4 Hz, Ar**H**), 7.54 (1H, *d*, *J* = 8 Hz, Ar**H**), 3.37–3.35 (4H, *m*, –C**H**_2_NC**H**_2_–), 1.85 (4H, *m*, –C**H**_2_C**H**_2_–). ^13^C NMR (Bruker, 100 MHz, CDCl_3_, δ_C_, ppm): 148.4, 133.5, 132.1, 131.5, 130.6, 123.9 (Ar**H**), 48.2 (–**C**H_2_N**C**H_2_–), 25.9 (–**C**H_2_ **C**H_2_–).

*2.2.1.2. N-Piperidinyl-o-nitro­benzene­sul­fonamide, **2**. o*-Nitro­benzene­sul­fonyl chloride (5.00 g, 22.57 mmol) and piperidine (3.84 g, 4.45 ml, 45.1 mmol). Yellow crystals (4.97 g, 81.5%); *R*_F_ = 0.56 (CH_2_Cl_2_/*n*-hexane, 2:1 *v*/*v*); m.p. 91.6–91.8 °C. IR (Bruker, ATR, ν, cm^−1^): 3076 (aryl C—H str.), 2947 (*sp* ^3^-C—H str.), 1552 (aryl C=C str.), 1550 (asym C—NO_2_ str.), 1354 (sym C—NO_2_ str.), 1350 (asym SO_2_—N str.), 1166 (sym SO_2_—N str.), 1056 (C—N str.). ^1^H NMR (Bruker, 400 MHz, CDCl_3_, δ_H_, ppm): 7.96 (1H, *d*, *J* = 4 Hz, Ar**H**), 7.70 (2H, *t*, *J* = 4 Hz, Ar**H**), 7.59 (1H, *d*, *J* = 8 Hz, Ar**H**), 3.26–3.24 (4H, *m*, –C**H**_2_NC**H**_2_–), 1.64–1.63 (4H, *m*, –C**H**_2_CH_2_C**H**_2_–), 1.55–1.54 (2H, *m*, –CH_2_C**H**_2_CH_2_–). ^13^C NMR (Bruker, 100 MHz, CDCl_3_, δ_C_, ppm): 148.5, 133.6, 131.6, 131.5, 130.8, 123.5 (Ar**H**), 47.0 (–**C**H_2_N**C**H_2_–), 25.4 (–**C**H_2_CH_2_ **C**H_2_–), 23.5 (–CH_2_ **C**H_2_CH_2_–).

*2.2.1.3. N-Indolinyl-o-nitro­benzene­sul­fonamide, **3**. o*-Nitro­benzene­sul­fonyl chloride (3.00 g, 13.54 mmol) and indoline (3.55 g, 3.34 ml, 29.79 mmol). Yellow crystals (3.11 g, 75.5%); *R*_F_ = 0.79 (CH_2_Cl_2_/*n*-hexane, 2:1 *v*/*v*); m.p. 106.5–106.8 °C. IR (Bruker, ATR, ν, cm^−1^): 3077 (aryl C—H str.), 2976 (*sp* ^3^-C—H str.), 1594 (aryl C=C str.), 1536 (asym C—NO_2_ str.), 1356 (sym C—NO_2_ str.), 1355 (asym SO_2_—N str.), 1161 (sym SO_2_—N str.), 1051 (C—N str.). ^1^H NMR (Bruker, 400 MHz, CDCl_3_, δ_H_, ppm): 7.95 (1H, *d*, *J* = 8 Hz, Ar**H**), 7.71 (1H, *t*, *J* = 8 Hz, Ar**H**), 7.62 (2H, *t*, *J* = 8 Hz, Ar**H**), 7.48 (1H, *d*, *J* = 8 Hz, Ar**H**), 7.21 (2H, *t*, *J* = 8 Hz, Ar**H**), 7.05 (1H, *t*, *J* = 8 Hz, Ar**H**), 4.17 (2H, *t*, *J* = 8 Hz, –NC**H**_2_–), 3.10 (2H, *t*, *J* = 8 Hz, –NCH_2_C**H**_2_–). ^13^C NMR (Bruker, 100 MHz, CDCl_3_, δ_C_, ppm): 148.4, 141.1, 134.1, 131.8, 131.7, 131.6, 130.2, 127.8, 125.5, 124.3, 124.2, 114.5 (**Ar**H), 50.5 (–N**C**H_2_–), 28.0 (–NCH_2_ **C**H_2_–).

#### *N*-Cyclo­amino-*o*-sul­fan­il­amides 4–6

An evacuated nitro­gen-gas-filled round-bottomed flask was charged with *N*-cyclo­amino-*o*-nitro­benzene­sul­fonamides **1**–**3** (15.63 mmol) dissolved in ethanol (30 ml), at ambient tem­per­ature, and 10% palladium-on-charcoal catalyst (3.35 mol%) was added, with stirring. Hydrogen gas was then introduced *via* a balloon and stirring continued at ambient tem­per­ature for 12 h. The reaction mixture was filtered and the solvent was evaporated *in vacuo*. The resulting residue was extracted into di­chloro­methane (50 ml), dried over anhydrous sodium sulfate, filtered and concentrated under reduced pressure to afford an oil, which was purified on a silica-gel column using di­chloro­methane and *n*-hexane (2:1 *v*/*v*). Crystals were obtained *via* slow solvent evaporation of the eluates at ambient tem­per­ature.

*2.2.2.1. N-Pyrrolidinyl-o-sul­fan­il­amide, **4**. N*-Pyrrolidinyl-*o*-nitro­benzene­sul­fonamide **1** (4.00 g, 15.63 mmol) with 10% palladium-on-charcoal catalyst (0.56 g, 5.26 mmol). Off-white crystals (2.90 g, 82%); *R*_F_ = 0.40 (CH_2_Cl_2_/*n*-hexane, 2:1 *v*/*v*); m.p. 75.2–75.4 °C. IR (Bruker, ATR, ν, cm^−1^): 3464, 3363 (N—H str.), 3003 (aryl C—H str.), 2947 (*sp* ^3^-C—H str.), 1620 (aryl C=C str.), 1323 (asym SO_2_—N str.), 1132 (sym SO_2_—N str.), 1307 (C—N str.). ^1^H NMR (Bruker, 400 MHz, CDCl_3_, δ_H_, ppm): 7.63 (1H, *d*, *J* = 8 Hz, Ar**H**), 7.28 (1H, *t*, *J* = 8 Hz, Ar**H**), 6.74 (2H, *d*, *J* = 8 Hz, Ar**H**), 5.13 (2H, *s*, NH), 3.31 (4H, *m*, –C**H**_2_NC**H**_2_–), 1.80 (4H, *m*, –C**H**_2_C**H**_2_–). ^13^C NMR (Bruker, 100 MHz, CDCl_3_, δ_C_, ppm): 146.4, 134.0, 130.2, 119.1, 117.6, 117.1 (**Ar**H), 47.8 (–**C**H_2_N**C**H_2_–), 25.2 (–**C**H_2_ **C**H_2_–).

*2.2.2.2. N-Piperidinyl-o-sul­fan­il­amide, **5**. N*-Piperidinyl-*o*-nitro­benzene­sul­fonamide **2** (4.00 g, 14.81 mmol) with 10% palladium-on-charcoal catalyst (0.53 g, 4.98 mmol). Off-white crystals (3.06 g, 86%); *R*_F_ = 0.57 (CH_2_Cl_2_/*n*-hexane, 2:1 *v*/*v*); m.p. 76.6–76.8 °C. IR (Bruker, ATR, ν, cm^−1^): 3487; 3383 (N—H str.), 3072 (aryl C—H str.), 2947 (*sp* ^3^-C—H str.), 1606 (aryl C=C str.), 1309 (asym SO_2_—N str.), 1136 (sym SO_2_—N str.), 1336 (C—N str.). ^1^H NMR (Bruker, 400 MHz, CDCl_3_, δ_H_, ppm): 7.48 (1H, *d*, *J* = 8 Hz, Ar**H**), 7.21 (1H, *t*, *J* = 8 Hz, Ar**H**), 6.67 (1H, *d*, *J* = 8 Hz, Ar**H**), 6.64 (1H, *d*, *J* = 8 Hz, Ar**H**), 4.99 (2H, *s*, NH), 3.03–3.00 (4H, *m*, –C**H**_2_NC**H**_2_–), 1.56–1.53 (4H, *m*, –C**H**_2_CH_2_C**H**_2_–), 1.39–1.37 (2H, *m*, –CH_2_C**H**_2_CH_2_–). ^13^C NMR (Bruker, 100 MHz, CDCl_3_, δ_C_, ppm): 146.3, 134.0, 130.1, 118.0, 117.6, 117.0 (**Ar**H), 46.8 (–**C**H_2_N**C**H_2_–), 25.2 (–**C**H_2_CH_2_ **C**H_2_–), 23.6 (–CH_2_ **C**H_2_CH_2_–).

*2.2.2.3. N-Indolinyl-o-sul­fan­il­amide, **6**. N*-Indolinyl-*o*-nitro­benzene­sul­fonamide **3** (2.50 g, 8.22 mmol) with 10% palladium-on-charcoal catalyst (0.29 g, 2.73 mmol). Off-white crystals (1.69 g, 75%); *R*_F_ = 0.80 (CH_2_Cl_2_/*n*-hexane, 2:1 *v*/*v*); m.p.: 111.9–112 °C. IR (Bruker, ATR, ν, cm^−1^): 3448, 3367 (N—H str.), 3070 (aryl C—H str.), 2924 (*sp* ^3^-C—H str.), 1597 (aryl C=C str.), 1327 (asym SO_2_—N str.), 1138 (sym SO_2_—N str.), 1330 (C—N str.). ^1^H NMR (Bruker, 400 MHz, CDCl_3_, δ_H_, ppm): 7.48 (2H, *d*, *J* = 8 Hz, Ar**H**), 7.16 (1H, *t*, *J* = 8 Hz, Ar**H**), 7.08 (1H, *t*, *J* = 6 Hz, Ar**H**), 7.03 (1H, *d*, *J* = 8 Hz, Ar**H**), 6.90 (1H, *t*, *J* = 8 Hz, Ar**H**), 6.58 (1H, *d*, *J* = 8 Hz, Ar**H**), 6.55 (1H, *d*, *J* = 8 Hz, Ar**H**), 5.00 (2H, *s*, NH), 3.96 (2H, *t*, *J* = 8 Hz, –NC**H**_2_–), 2.86 (2H, *t*, *J* = 8 Hz, –NCH_2_C**H**_2_–). ^13^C NMR (Bruker, 100 MHz, CDCl_3_, δ_C_, ppm): 146.4, 142.3, 134.4, 132.1, 129.8, 127.6, 125.1, 123.7, 119.4, 117.7, 117.3, 115.1 (**Ar**H), 50.0 (–N**C**H_2_–), 28.1 (–NCH_2_ **C**H_2_). Reaction synthesis of nitro­sul­fonamides **1**–**3** and amino­sul­fonamides **4**–**6** are presented in Scheme S1 in the supporting information. The FT–IR, MS and ^1^H/^13^C NMR spectra of com­pounds **1**–**6** are also presented in the supporting information.

### Docking studies

#### Preparation of the ligands for docking

The X-ray crystal structures of synthesized com­pounds **1**–**6** (CIF files) were imported directly into the *Schrödinger Suite* (Schrödinger, 2022 ▸) for preparation. The reference drugs *N*-(5-sulfamoyl-1,3,4-thia­di­azol-2-yl)-2-(thio­phen-2-yl)acetamide (**A**) and 5-[1-(naph­tha­len-1-yl)-1,2,3-tri­azol-4-yl]thio­phene-2-sul­fon­amide (**B**) were based on chemical structures downloaded from the *PubChem* (https://pubchem.ncbi.nlm.nih.gov/) web­site in SDF format. **A** and **B** were used as reference com­pounds because they are natural ligands in the crystalline state of 5fl4 and 4iwz. The *Ligprep* module of the mol­ecular model­ling platform of the *Schrödinger Suite* (Schrödinger, 2022 ▸) was then used to prepare the imported structures by assigning bond lengths, bond angles, generating possible ionization states at pH 7 using *Epik* and finally to optimize using the OPLS4 force field (Nainwal *et al.*, 2018 ▸).

#### Protein preparation

The protein structures of 4iwz and 5fl4, with resolutions of 1.60 and 1.82 Å, respectively, were downloaded from the Research Collaboratory for Structural Bioinformatics (RCSB) Protein Data Bank (PDB). Retrieved crystal coordinates were prepared in the ‘Protein Preparation Wizard’ of the *Schrödinger Suite* (Schrödinger, 2022 ▸), with default parameters of assigning bond orders, optimizing and minimization using OPLS4. A receptor grid generation mod­ule was applied to the prepared proteins by selecting the corresponding cocrystallized ligand to define the binding site. A default parameter for the radii of van der Waals having a scaling factor of 1 Å with a partial charge cut-off of 0.25 Å was used (Panwar & Singh, 2021 ▸; Yang *et al.*, 2022 ▸).

#### Mol­ecular docking

Docking calculations were executed in the extra precision (XP) mode of the *Glide* module in the mol­ecular modelling platform of the *Schrödinger Suite* (Schrödinger, 2022 ▸). The com­plexes with the highest negative docking scores have better binding towards the respective proteins 4iwz and 5fl4. Docking calculations of the synthesized *N*-cyclo­amino derivatives against the hCA II (PDB entry 5fl4) and XII (4iw7) isoforms will provide a selectivity profile that may be inter­esting for the development of novel anti­cancer agents with limited side effects. The hCA II (PDB entry 5fl4) and XII (4iw7) carbonic anhydrase iso­forms have recently emerged as excellent targets for the design of novel therapeutic strategies for cancer, due to their involvement in the survival of tumour cells, as well as in the insurgence of resistance to classical anti­cancer protocols (Milite *et al.*, 2019 ▸).

### DFT calculations

Theoretical studies were performed for com­pounds **1**–**6** whereupon the SC-XRD structures of the com­pounds were used for optimization and global reactivity descriptor (GRD) calculations. Computational studies and mol­ecular electrostatic potential (MEP) for **1**–**6** were carried out using the *GAUSSIAN16* software package (Frisch *et al.*, 2016 ▸), whereas the calculations were performed using the standard hybrid density functional method (B3LYP) with a basis set of the 6-311++G**(p,d) level (Becke, 1993 ▸). Optimized mol­ecules were obtained with the *Chemcraft* visualization program (https://www.chemcraftprog.com/).

### Refinement

Crystal data, data collection and structure refinement details are summarized in Table 1 ▸. Carbon-bound H atoms were added in idealized geometrical positions in a riding model. Nitro­gen-bound H atoms were located in a difference map and refined freely.

## Results and discussion

### Chemistry

The *N*-cyclo­amino-*o*-sul­fan­il­amides **4**–**6** were prepared *via* a two-step reaction, starting from the condensation reaction of *o*-nitro­benzene­sul­fonyl chloride with alicyclic amines in tolu­ene, at ambient tem­per­ature, to afford *N*-cyclo­amino-*o*-nitro­benzene­sul­fonamide adducts **1**–**3** (Scheme S1 in the supporting information). The use of toluene as a nonpolar reaction medium was, amongst other reasons, to drive the forward reaction. In the second step, adducts **1**–**3** were hydro­genated with hydro­gen gas, in ethanol at ambient tem­per­ature, in the presence of 10% palladium-on-activated charcoal catalyst to give the target *N*-cyclo­amino-*o*-sul­fan­il­amides **4**–**6** in 72–86% yield. The reactions were monitored by TLC.

All the com­pounds synthesized were characterized by their melting points and IR, ^1^H/^13^C NMR and MS spectra. In the IR spectra of *o*-nitro­sul­fonamide adducts **1**–**3**, the strong absorption bands observed at 1355–1342 and 1171–1161 cm^−1^ were ascribed to the asymmetric and symmetric stretching frequencies, respectively, of the SO_2_—N moiety, thereby alluding to the formation of the sul­fonamide bond. The disappearances of the SO_2_—Cl (1420 and 1220 cm^−1^) and N—H (3286–3265 cm^−1^) stretching bands in the IR spectra of *o*-nitro­benzene­sul­fonyl chloride and cyclo­amines, respectively, were good indicators of a successful condensation reaction. This was corroborated by the shift of the sul­fonyl (–SO_2_–) absorption bands from 1420 and 1220 (in *o*-nitro­benzene­sul­fonyl chloride) to 1355–1342 and 1171–1161 cm^−1^ (in **1**–**3**). It is noteworthy that the lower wavenumbers observed in the IR spectra of *o*-nitro­sul­fonamides **1**–**3** for –SO_2_– were not unusual as the Cl atom bonded to it had been replaced by a less electronegative N atom. In the IR spectra of *o*-sul­fan­il­amides **4**–**6**, the appearance of two N—H stretching bands in the higher frequency region around 3467 ± 20 and 3383 ± 10 cm^−1^, and the disappearance of the nitro (NO_2_) absorption bands (observed at 1550–1536 and 1369–1342 cm^−1^) in the spectra of **1**–**3** were attributed to the successful catalytic reduction of the nitro group to the amino group.

The ^1^H NMR spectra of *o*-nitro­sul­fonamides **1**–**3** were additive of the individual spectra of the starting materials (*i.e. o*-nitro­benzene­sul­fonyl chloride and cyclo­amines), with the disappearance of the nitro­gen proton peaks of cyclo­amines. The aromatic protons of *o*-sul­fan­il­amides **4**–**6** resonated upfield in com­parison to the same aromatic protons in precursors **1**–**3**. This general shift towards tetra­methyl­silane (TMS) was credited to the newly formed amino groups whose lone-pair electrons are suspected of having caused the increased mesomeric shielding of the aromatic protons. D_2_O-exchangeable singlets were also observed in the ^1^H NMR spectra of **4**–**6** between 5.13 and 4.99 ppm for the newly-formed amino protons. The success of the catalytic hydro­genation of nitro adducts **1**–**3** was corroborated by the ^13^C NMR spectra of **4**–**6**, where the requisite C atoms (C—NO_2_ → C—NH_2_) resonated upfield in the range 133.9–130.1 ppm. The spectroscopic data analyses of the synthesized com­pounds were consistent with the assigned structures of the com­pounds.

### Crystal structure

**Figure 2:**
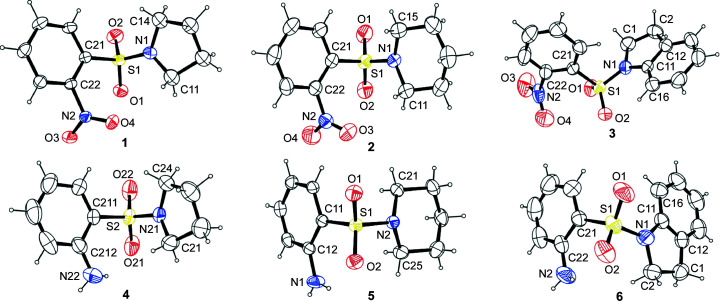
The mol­ecular structures of *o*-nitro­sul­fonamides **1**–**3** and *N*-cyclo­amino-*o*-sul­fan­il­amides **4**–**6** (molecule 2 of **4** shown). Displacement ellipsoids are drawn at the 50% probability level. Minor disorder com­ponents have been omitted.

The mol­ecules of **1**–**3** and **4**–**6** crystallized in the monoclinic space group *P*2_1_/*n* or *P*2_1_/*c* (No. 14), except for **5**, which crystallized in the ortho­rhom­bic space group *Pbca* (No. 61). In addition, they all had one mol­ecule in the asymmetric unit, with the exception of **4**, with two independent mol­ecules per asymmetric unit cell. The two mol­ecules per unit cell of com­pound **4** were identical but for the conformation of the pyrrolidine group (*cf.* Fig. S1 in the supporting information). It is noteworthy that the pyrrolidine ring in **1** is disordered. The mol­ecular structures of **1**–**3** and **4**–**6** are shown in Fig. 2 ▸, while the crystal data collection parameters of *o*-nitro­sul­fonamides **1**–**3** and *N*-cyclo­amino-*o*-sul­fan­il­amides **4**–**6** are presented in Table 1 ▸. They are com­pared with the crystal structure data of *para*-sul­fan­il­amide and *ortho*-sul­fan­il­amide, which crystallize in the ortho­rhom­bic *Pbca* (No. 61) and monoclinic *P*2_1_/*c* (No. 14) space groups, respectively (Gelbrich *et al.*, 2008 ▸; Shad *et al.*, 2008 ▸). Several sul­fonamide derivatives have also been reported (El-Gaby *et al.*, 2020 ▸). Selected bond lengths and angles, as determined from the SC-XRD experiments, are collected in Table S1 (see supporting information).

**Figure 3:**
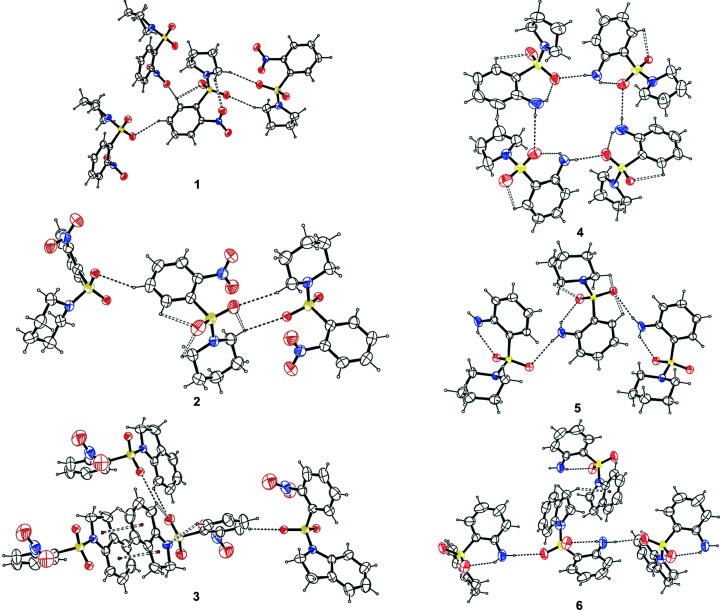
Selected hydro­gen-bond, C—H⋯(π ring) and π–π stacking inter­actions for com­pounds **1**–**6**. Displacement ellipsoids are drawn at the 50% probability level. Minor disorder com­ponents have been omitted.

It is instructive to note that the amino (NH_2_) group in *N*-cyclo­amino-*o*-sul­fan­il­amides **4**–**6** contributed significantly to their hydro­gen-bond inter­actions (*cf.* Table 2 ▸). In all three structures, there were intra­molecular N—H⋯O=S inter­actions resulting in ring closures that can be described with *S*(6) graph-set descriptors (Bernstein *et al.*, 1995 ▸; Etter *et al.*, 1990 ▸). Furthermore, com­pounds **5** and **6** exhibited infinite-chain inter­molecular N—H⋯O=S inter­actions with *C*(6) descriptors. Inter­estingly, no infinite chain inter­action was observed in **4**; instead, four mol­ecules were linked into a ring structure with an 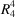 (24) descriptor. The *p*-sul­fan­il­amide (Gelbrich *et al.*, 2008 ▸) and *o*-sul­fan­il­amide (Shad *et al.*, 2008 ▸) structures also each have a number of infinite-chain inter­actions and ring structures. Fig. 3 ▸ shows selected hydro­gen-bond, C—H⋯(π ring) and π–π stacking inter­actions for sul­fonamides **1**–**3** and sul­fan­il­amides **4**–**6**. All the hydro­gen bonds were of moderate (mostly electrostatic) strength (Jeffrey, 1997 ▸), with **4** giving the strongest hydro­gen bonds (Table 2 ▸). Additionally, the com­pounds also exhibited both intra- and inter­molecular C—H⋯O=S inter­actions, with the length of the shortest inter­actions varying in the range 2.30–2.48 Å.

The only π–π stacking inter­action of note occurred in **3**, where two centroid-to-centroid inter­actions with distances of 3.6967 (11) Å were observed between the centrosymmetric indo­line moieties. An N=O⋯π ring inter­action of 3.657 (2) Å was also evident in **3**, whereas inter­molecular C—H⋯(π ring) inter­actions of 2.97 Å and S=O⋯(π ring) inter­actions of 3.5773 (15) Å were present in the structure of its hydro­genated analogue **6**. The packing diagrams of the crystal structures of com­pounds **1**–**6** are shown in Fig. S2 in the supporting information.

### Hirshfeld surface analysis

The Hirshfeld surface analyses (Turner *et al.*, 2017 ▸) of com­pounds **1**–**6** showed inter­molecular inter­actions such as O—H⋯O, O—H⋯N and C—H⋯π. Two sharp O—H spikes typical of an O—H⋯O inter­action from **1** contributed the highest O⋯H inter­action of 42.3%. The fingerprint plots showed that C⋯H contacts were highest for **6** (30.4%), and this is closely related to C—H⋯π inter­actions (McKinnon *et al.*, 2007 ▸; Kolade *et al.*, 2020 ▸). The percentages of the major contributions, *e.g.* C⋯H, O⋯H and N⋯H inter­atomic contacts, for each mol­ecule are com­piled in Table 3 ▸.

The mol­ecular Hirshfeld surfaces, mapped as *d*_norm_, shape index and curvedness, confirmed inter­actions between neighbouring mol­ecules of **1**–**6** and are presented in Fig. S3. The large circular depressions (deep red) visible on the *d*_norm_ surfaces typically indicate that the mol­ecule has a donor site(s) (*e.g.* amine and/or sul­fone) or inter­actions with proteins.

Fingerprint plots of *o*-nitro­sul­fonamides **1**–**3** and *N*-cyclo­amino-*o*-sul­fan­il­amides **4**–**6** in full and resolved into C⋯H, O⋯H and N⋯H are presented in Fig. S4 (supporting information). The inter­molecular O⋯H and N⋯H inter­actions appear as two distinct spikes of almost equal length in the 2D (two-dimensional) fingerprint plots in the region 1.2 < (*d*_e_ + *d*_i_) < 2.9 Å as light-sky-blue patterns in full fingerprint 2D plots and characterized to be 2.56 ± 0.21 Å corresponds to O⋯H contacts which contributes the majority of the surface area. 2D fingerprint plots reveal the contributions of these inter­actions in the crystal structure qu­anti­tatively and are presented in Table 4 ▸ (with minimum and maximum values of *d*_norm_, *d*_i_ and *d*_e_ provided). Complementary regions are also visible in the fingerprint plots (Fig. S4), where one mol­ecule acts as a donor (*d*_e_ > *d*_i_) and the other acts as an acceptor (*d*_e_ < *d*_i_). This finding was validated by the calculated mol­ecular electrostatic potential of **1**–**6** (Fig. S5). The negative potential (acceptor) is indicated as a red surface around the O atoms attached to sulfur (–SO_2_) and the N atoms attached to oxygen (–NO_2_). The blue/purple surface area indicates that the positive potential (donor) is mapped in the proximity of the H atoms (Fig. S5).

### Global reactivity descriptors (GRDs)

The full geometry of optimized mol­ecules **1**–**6** presented bond lengths similar to those obtained from the crystal data. A com­parison of selected torsion angles of the crystal structures of **1**–**6** and the DFT-optimized mol­ecules showed that con­formation of the mol­ecules did not change significantly in the DFT-optimized state (Fig. S6). Generally, the observed, almost flat, O—S—N—C torsion angle of the DFT-optimized mol­ecules suggest that the lone pairs on sulfur may have con­tributed to the π-electron delocalization that is observed in the DFT mol­ecules.

**Figure 4:**
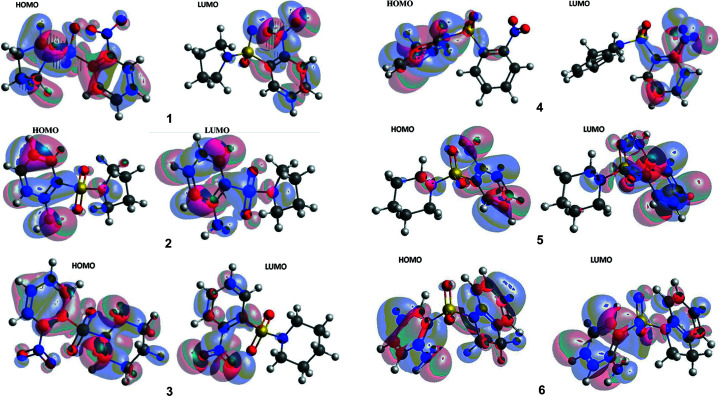
Frontier mol­ecular orbitals for the optimized structures of **1**–**6**.

The highest occupied mol­ecular orbital (HOMO) and lowest unoccupied mol­ecular orbital (LUMO) electrons are distributed around various moieties within the various mol­ecules (Fig. 4 ▸). Generally, electron distribution is mainly scattered in the HOMO over the phenyl, sulfur and indolin­yl/pyrrolidinyl rings, with the exception of **3** and **5**. The LUMO is mainly spread over the phenyl moieties. This indicates that there is a transfer of charge between the indolin­yl/pyrrolidinyl rings and the phenyl moieties within the mol­ecule.

The HOMO–LUMO gap, which describes the stability of mol­ecules and predicts reactivity between species by providing the electrical transport properties, as well as electron carrier and mobility in mol­ecules (Rathi *et al.*, 2020 ▸), are provided in Table 5 ▸. *N*-Indolinyl-*o*-nitro­benzene­sul­fonamide **3** displayed the smallest energy gap (3.24 eV), indicating that it was the softest mol­ecule with good polarizability and reactivity, whereas *N*-piperidinyl-*o*-sul­fan­il­amide **5** presented the largest energy gap of 4.924 eV, thereby corroborating its high chemical hardness of 2.462 eV (*cf*. Table 5 ▸). The lowest LUMO energy was obtained from **3** (*E*_LUMO_ = −3.175 eV), indicating that it is the best electron acceptor of the mol­ecules analyzed, whereas **6** was the best electron donor in the series, with the highest HOMO energy (*E*_HOMO_) of −6.142 eV (Table 5 ▸). The observed large energy gap (4.924 eV) in **5** suggests that charge transfer could promote its bioactivity and ability to form biological inter­actions at the piperidinyl and phenyl moiety (Al-Wahaibi *et al.*, 2019 ▸). Therefore, the predicted order of biological inter­actions are **5** > **6** > **4** > **2** > **1** > **3**.

The ionization potential (*I*), electron affinity (*A*), chemical potential (μ), electronegativity (χ), global hardness (η), global softness (*S*) and global electrophilicity (ω) values were calculated using the HOMO and LUMO energy values and are collated in Table 5 ▸. The lowest *I* value of 6.142 eV originated from sul­fan­il­amide **6**, whereas sul­fonamide **3** gave the largest *A* value of 3.175 eV. Amongst the com­pounds studied, **2** gave the highest χ value of 5.1795 eV. Inter­estingly, sul­fan­il­amide **5** displayed the highest η value of 2.462 eV and the lowest chemical softness (*S*) of 0.406 eV, thus alluding to its having the most reactive nature of all the mol­ecules investigated. The highest global electrophilicity of 29.597 eV was also recorded for sul­fonamide **2**, indicating that it is a strong electro­phile. In general, the chemical reactivities of com­pounds **1**–**6** have been shown to vary with the groups attached to the com­pounds (Abbaz *et al.*, 2018 ▸).

### Docking studies

**Figure 5:**
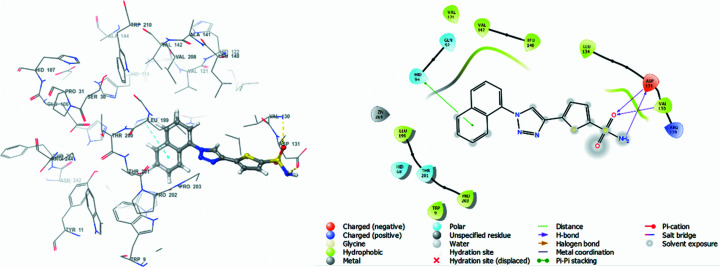
(*a*) 3D inter­action diagram of *N*-(5-sulfamoyl-1,3,4-thia­di­azol-2-yl)-2-(thio­phen-2-yl)acetamide (**A**) and hCA II isoenzyme 4iwz (**A**), and (*b*) 2D inter­action diagram depicting the binding residues of 4iwz.

Docking studies of synthesized **1**–**6** with human carbonic anhydrase II and IX inhibitors (hCA II and IX; PDB entries: 4iwz and 5fl4) (Biswas *et al.*, 2013 ▸; Leitans *et al.*, 2015 ▸), downloaded from the Research Collaboratory for Structural Bioinformatics (RCSB) Protein Data Bank (PDB) was carried out in *Maestro* (Version 13.1.137, MMshare Version 5.7.137, Release 2022-1, Platform Windows-x64) (Schrödinger, 2022 ▸). The binding strengths of the docked com­plexes were analysed through docking score, glide E-model and ligand efficiency (*cf*. Table 6 ▸). These energies define the degree of stability of binding between the respective isoenzymes and target com­pounds **1**–**6**. *N*-(5-Sulfamoyl-1,3,4-thia­di­azol-2-yl)-2-(thio­phen-2-yl)acetamide (**A**) and 5-[1-(naph­tha­len-1-yl)-1,2,3-tri­azol-4-yl]thio­phene-2-sul­fonamide (**B**) were also docked with respective proteins 4iwz and 5fl4, and taken as reference or standard drugs. Docking poses for the synthesized com­pounds are displayed in Figs. S7–S18, while those for the reference drugs are shown in Figs. 5 ▸ and 6 ▸.

Docking calculations between 4iwz and **A** (reference drug) displayed a docking score of −2.252 kcal mol^−1^, which is higher than for all synthesized com­pounds **1**–**6** (*cf*. Table 6 ▸).

Also, **A** inter­acted with amino acid residues GLN_92_ (2.39 Å) and HIE_64_ (2.24 Å) *via* hydro­gen-bonding inter­actions and with amino acid residue HIS_94_ (4.75 Å) *via* π–π stacking inter­actions (*cf*. Table 7 ▸). Some bad inter­actions/contacts were observed between the amino acid residue GLU_106_ and **A** (Fig. 5 ▸). Compound **2** displayed the best binding affinity among the synthesized com­pounds, with a docking score of −2.223 kcal mol^−1^, slightly lower than that of the reference drug. Sulfanilamides **4** and **5** also displayed significantly good binding affinities, with docking scores of −1.645 and −1.636 kcal mol^−1^, respectively. Sulfanilamide **6** was characterized by the lowest binding affinity, evidenced by its docking score of −0.784 kcal mol^−1^. Compound **6** displayed glide a E-model energy of −47.945 kcal mol^−1^ and a ligand efficiency of −0.041 kcal mol^−1^. Structurally, sul­fan­il­amide **6** inter­acted with the protein 4iwz through hydro­gen bonding with GLN_92_ (1.88 Å) and TRP_5_ (2.11 Å), and through π–π stacking with THR_199_ (1.81 Å) (Fig. S12).

**Figure 6:**
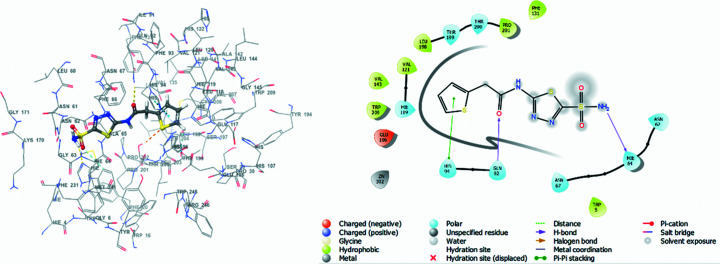
(*a*) 3D inter­action diagram of 5-[1-(naph­tha­len-1-yl)-1,2,3-tri­azol-4-yl]thio­phene-2-sul­fonamide (**B**) and hCA IX isoenzyme 5fl4, and (*b*) 2D inter­action diagram depicting the binding residues of 5fl4.

To determine the mode of inter­action of the synthesized com­pounds with human carbonic anhydrase IX inhibitor (hCA IX), the synthesized com­pounds were docked into the active site of 5fl4, and the results obtained were com­pared with the docked results of the reference drug **B**. We observed that the reference drug inter­acts with amino acid residues ASP_13_ (1.59–2.73 Å) and VAL_130_ (2.53 Å) *via* hydro­gen bonding, and with HID_94_ (5.49 Å) *via* π–cation inter­actions (*cf*. Table 7 ▸). Furthermore, **B** exhibited a docking score of −1.969 kcal mol^−1^, a glide E-model energy of −41.029 kcal mol^−1^ and a ligand efficiency of −0.082 kcal mol^−1^, and is surrounded by several amino acid residues. Some of the residues are TRP_9_, PRO_203_, THR_201_, HID_68_, LEU_199_, HID_94_, GLN_92_, VAL_171_ and ZN_264_, with bad contacts or inter­actions observed on residue ASP_131_ (Fig. 6 ▸). Benzene­sul­fonamide **2** presented the highest binding affinity, with a docking score of −1.977 kcal mol^−1^, higher than the reference drug. All other synthesized com­pounds, except for *N*-cyclo­amino-*o*-nitro­benzene­sul­fonamide **1** (docking score = −0.807), displayed significantly good docking scores; however, they were lower than the reference drug (*cf*. Table 6 ▸). Com­pound **3** displayed hydro­gen-bond inter­actions with amino acid residue GLN_71_, with a bond length of 2.26 Å, and a π–cation inter­action with amino acid residue HID_94_, with a bond length of 4.05 Å (Fig. S15).

We observed that the docking scores of **2** with 4iwz and 5fl4 are close to those obtained for **A** with 4iwz and **B** with 5fl4. Docking scores of mol­ecules with ring structures **1** and **3**–**6** (in the range > −1.67 kcal mol^−1^) also correlated with the electronegativity and electrophilicity values presented in Table 5 ▸. This is informed by the HOMO and LUMO properties (Kumar *et al.*, 2018 ▸).

## Conclusion

*o*-Nitro­sul­fonamides **1**–**3** and *N*-cyclo­amino-*o*-sul­fan­il­amides **4**–**6** have been successfully synthesized, characterized and the intermolecular interactions analysed, as well as being tested *in silico* for carbonic anhydrase II (4iwz) and IX (5fl4) inhibitory activities. The results obtained from crystal packing and DFT analysis suggests that the mol­ecules are held together by forces such as hydro­gen bonding and π–π inter­actions. The results of the DFT study of com­pounds **1**–**6** were correlated with the mol­ecular docking data and indicate that electronegativity and electrophilicity of the title com­pounds play an important role in their inter­action with carbonic anhydrase II (4iwz) and IX (5fl4).

*O*-Nitro­sul­fonamide **2** displayed a good docking score against 4iwz (lower than the reference drug) and the best against 5fl4 (higher than the reference drug). These results provided a valuable synthesis approach and structural and docking information for com­pounds **1**–**6** that may be used for the development of potent anti­bacterial drugs.

## Supplementary Material

Crystal structure: contains datablock(s) ka097, ja198, ja250, ja192, ka115, ja189, global. DOI: 10.1107/S2053229622010130/oj3005sup1.cif

Structure factors: contains datablock(s) ka097. DOI: 10.1107/S2053229622010130/oj3005ka097sup2.hkl

Structure factors: contains datablock(s) ja198. DOI: 10.1107/S2053229622010130/oj3005ja198sup3.hkl

Structure factors: contains datablock(s) ja250. DOI: 10.1107/S2053229622010130/oj3005ja250sup4.hkl

Structure factors: contains datablock(s) ja192. DOI: 10.1107/S2053229622010130/oj3005ja192sup5.hkl

Structure factors: contains datablock(s) ka115. DOI: 10.1107/S2053229622010130/oj3005ka115sup6.hkl

Structure factors: contains datablock(s) ja189. DOI: 10.1107/S2053229622010130/oj3005ja189sup7.hkl

Supporting information file. DOI: 10.1107/S2053229622010130/oj3005ka097sup8.cml

Supporting information file. DOI: 10.1107/S2053229622010130/oj3005ja198sup9.cml

Supporting information file. DOI: 10.1107/S2053229622010130/oj3005ja250sup10.cml

Supporting information file. DOI: 10.1107/S2053229622010130/oj3005ja192sup11.cml

Supporting information file. DOI: 10.1107/S2053229622010130/oj3005ka115sup12.cml

Supporting information file. DOI: 10.1107/S2053229622010130/oj3005ja189sup13.cml

Additional figures, tables and spectra. DOI: 10.1107/S2053229622010130/oj3005sup14.pdf

CCDC references: 2014232, 2039638, 2014231, 2014230, 2014229, 2039639

## Figures and Tables

**Table:** Experiments were carried out with Mo *K*α radiation using a Bruker APEXII CCD diffractometer. Absorption was corrected for by numerical methods (*SADABS*; Bruker, 2008 ▸).

	**1**	**2**	**3**
Crystal data			
Chemical formula	C_10_H_12_N_2_O_4_S	C_11_H_14_N_2_O_4_S	C_14_H_12_N_2_O_4_S
*M*_r_	256.28	270.30	304.32
Crystal system, space group	Monoclinic, *P*2_1_/*n*	Monoclinic, *P*2_1_/*n*	Monoclinic, *P*2_1_/*n*
Temperature (K)	200	296	296
*a*, *b*, *c* (Å)	8.6173 (5), 14.6662 (9), 9.4885 (6)	8.6881 (9), 15.0266 (14), 9.8337 (10)	7.4701 (5), 23.6743 (12), 7.8614 (5)
α, β, γ (°)	90, 108.075 (3), 90	90, 106.526 (4), 90	90, 94.989 (3), 90
*V* (Å^3^)	1140.01 (12)	1230.8 (2)	1385.02 (15)
*Z*	4	4	4
μ (mm^−1^)	0.29	0.27	0.25
Crystal size (mm)	0.67 × 0.67 × 0.12	0.48 × 0.47 × 0.45	0.62 × 0.51 × 0.43

Data collection			
*T*_min_, *T*_max_	0.934, 1.000	0.941, 1.000	0.913, 1.000
No. of measured, independent and observed [*I* > 2σ(*I*)] reflections	24329, 2849, 2508	25328, 3059, 2662	28400, 3434, 2864
*R*_int_	0.018	0.017	0.024
(sin θ/λ)_max_ (Å^−1^)	0.669	0.668	0.669

Refinement			
*R*[*F* ^2^ > 2σ(*F* ^2^)], *wR*(*F* ^2^), *S*	0.041, 0.111, 1.05	0.036, 0.106, 1.06	0.040, 0.105, 1.05
No. of reflections	2849	3059	3434
No. of parameters	149	163	190
No. of restraints	20	0	0
H-atom treatment	H-atom parameters constrained	H-atom parameters constrained	H-atom parameters constrained
Δρ_max_, Δρ_min_ (e Å^−3^)	0.43, −0.47	0.32, −0.31	0.26, −0.29

**Table:** 

	**4**	**5**	**6**
Crystal data			
Chemical formula	C_10_H_14_N_2_O_2_S	C_11_H_16_N_2_O_2_S	C_14_H_14_N_2_O_2_S
*M*_r_	226.29	240.32	274.33
Crystal system, space group	Monoclinic, *P*2_1_/*c*	Orthorhombic, *P* *b* *c* *a*	Monoclinic, *P*2_1_/*n*
Temperature (K)	296	200	296
*a*, *b*, *c* (Å)	16.399 (3), 7.9485 (12), 18.376 (3)	11.1747 (4), 10.4850 (4), 20.1368 (8)	9.7990 (11), 10.2612 (13), 13.2010 (16)
α, β, γ (°)	90, 113.907 (6), 90	90, 90, 90	90, 100.682 (5), 90
*V* (Å^3^)	2189.7 (6)	2359.36 (15)	1304.4 (3)
*Z*	8	8	4
μ (mm^−1^)	0.28	0.26	0.25
Crystal size (mm)	0.47 × 0.32 × 0.20	0.49 × 0.26 × 0.25	0.54 × 0.34 × 0.34

Data collection			
*T*_min_, *T*_max_	0.927, 1.000	0.944, 1.000	0.925, 1.000
No. of measured, independent and observed [*I* > 2σ(*I*)] reflections	55955, 5477, 4489	57075, 2935, 2583	28600, 3275, 2726
*R*_int_	0.032	0.020	0.031
(sin θ/λ)_max_ (Å^−1^)	0.670	0.668	0.675

Refinement			
*R*[*F* ^2^ > 2σ(*F* ^2^)], *wR*(*F* ^2^), *S*	0.035, 0.107, 1.04	0.029, 0.087, 1.05	0.039, 0.113, 1.05
No. of reflections	5477	2935	3275
No. of parameters	287	153	180
No. of restraints	0	0	0
H-atom treatment	H atoms treated by a mixture of independent and constrained refinement	H atoms treated by a mixture of independent and constrained refinement	H atoms treated by a mixture of independent and constrained refinement
Δρ_max_, Δρ_min_ (e Å^−3^)	0.33, −0.36	0.34, −0.36	0.27, −0.43

Computer programs: *APEX2* (Bruker, 2011 ▸), *SAINT* (Bruker, 2012 ▸), *SHELXT2018* (Sheldrick, 2015*a* ▸), *SHELXL2018* (Sheldrick, 2015*b* ▸), *ShelXle* (Hübschle *et al.*, 2011 ▸), *ORTEP-3 for Windows* (Farrugia, 2012 ▸), *PLATON* (Spek, 2020 ▸) and *Mercury* (Macrae *et al.*, 2020 ▸).

**Table 2:** Hydrogen-bond, C—H⋯(π ring) and π–π stacking inter­action geometry (Å, °) for the crystal structures of *p*-sul­fan­il­amide (**B**), *o*-sul­fan­il­amide (**D**), *o*-nitro­sul­fonamides **1**–**3** and *N*-cyclo­amino-*o*-sul­fan­il­amides **4**–**6**

Compound	Inter­action	*D*—H	H⋯*A*	*D*⋯*A*	*D*—H⋯*A*	π–π
*o*-Nitrosul­fonamides						
**1**	C11—H11*A*⋯O1^i^	0.99	2.56	3.398 (7)	143	
	C11—H11*B*⋯O4	0.99	2.48	3.093 (9)	120	
	C25—H25⋯O4^ii^	0.95	2.54	3.240 (2)	131	
	C25—H25⋯O1^iii^	0.95	2.47	3.229 (2)	137	
	C26—H26⋯O2	0.95	2.49	2.860 (2)	103	
	C26—H26⋯O3^iv^	0.95	2.56	3.467 (2)	160	
**2**	C11—H11*A*⋯O2	0.97	2.48	2.907 (2)	107	
	C11—H11*A*⋯O2^i^	0.97	2.59	3.476 (2)	152	
	C15—H15*B*⋯O1	0.97	2.51	2.943 (2)	107	
	C25—H25⋯O2^v^	0.93	2.57	3.334 (2)	140	
	C26—H26⋯O1	0.93	2.53	2.877 (2)	103	
**3**	C16—H16⋯O2	0.93	2.41	2.975 (2)	119	
	C16—H16⋯O2^vi^	0.93	2.55	3.195 (2)	127	
	C23—H23⋯O1^vii^	0.93	2.30	3.086 (3)	142	
	C26—H26⋯N1	0.93	2.60	2.983 (2)	105	
	*Cg*1⋯*Cg*2^i^					3.6967 (11)
	*Cg*2⋯*Cg*1^i^					3.6968 (11)
						
*N*-Cyclo­amino-*o*-sul­fan­il­amides						
**4**	N12—H12*C*⋯O21	0.78 (3)	2.40 (3)	3.121 (3)	155 (3)	
	N12—H12*D*⋯O11	0.84 (3)	2.10 (3)	2.776 (3)	138 (3)	
	N22—H21*C*⋯O11^i^	0.82 (3)	2.30 (3)	3.106 (2)	168 (2)	
	N22—H21*D*⋯O21	0.87 (2)	2.07 (2)	2.783 (2)	139 (2)	
	C114—H114⋯O22^viii^	0.93	2.59	3.391 (3)	144	
	C116—H116⋯O12	0.93	2.46	2.851 (2)	105	
	C216—H216⋯O22′	0.93	2.52	2.897 (2)	105	
**5**	N1—H1*A*⋯O1^ix^	0.836 (19)	2.496 (19)	3.2999 (16)	161.8 (16)	
	N1—H1*B*⋯O2	0.852 (16)	2.156 (16)	2.8240 (17)	135.1 (14)	
	C16—H16⋯O1	0.95	2.48	2.8774 (15)	105	
	C16—H16⋯O2^x^	0.95	2.48	3.2577 (15)	139	
	C21—H21*A*⋯O1	0.99	2.55	2.9635 (15)	105	
	C25—H25*B*⋯O2	0.99	2.44	2.8675 (16)	106	
**6**	N2—H2*C*⋯O2	0.86 (3)	2.19 (3)	2.857 (2)	134 (2)	
	N2—H2*D*⋯O1^xi^	0.89 (2)	2.22 (2)	3.098 (2)	168.8 (19)	
	C15—H15⋯O2^xii^	0.93	2.59	3.366 (2)	141	
	C16—H16⋯O1	0.93	2.58	3.105 (2)	117	
	C26—H26⋯O1	0.93	2.45	2.849 (2)	106	
	C1—H1*B*⋯*Cg*1^xiii^	0.97	2.97	3.817 (2)	147	
	S1—O2⋯*Cg*2^i^			3.5773 (15)		

Symmetry codes: (i) −*x* + 1, −*y* + 1, −*z* + 1; (ii) −*x* + 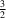 , *y* + 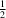 , *z* − 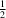 ; (iii) *x* + 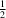 , −*y* + 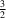 , *z* + 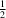 ; (iv) *x* + 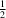 , −*y* + 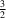 , *z* − 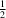 ; (v) *x* − 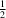 , −*y* + 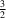 , *z* + 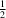 ; (vi) −*x* + 2, −*y* + 1, −*z* + 1; (vii) *x* + 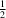 , −*y* + 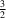 , *z* − 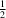 ; (viii) −*x* + 2, *y* − 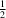 , −*z* + 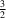 ; (ix) *x* − 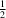 , *y*, −*z* + 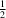 ; (x) −*x* + 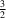 , *y* − 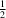 , *z*; (xi) *x* − 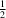 , −*y* + 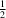 , *z* − 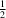 ; (xii) *x* − 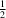 , −*y* + 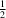 , *z* + 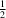 . Centroids for **3**: *Cg*1 N1/C1/C2/C12/C11; *Cg*2 C11–C16; *Cg*3 C21–C26; for **4**: *Cg*1 C22–C27; for **5** *Cg*1 C11–C16; *Cg*2 N1/C2/C1/C12/C11; for **6** *Cg*1 C21–C26.

**Table 3:** Percentage contributions of selected inter­atomic contacts to the Hirshfeld surface of com­pounds **1**–**6**

	C⋯H	O⋯H	N⋯H
*o*-Nitrosul­fonamides			
**1**	15.2	42.3	0.6
**2**	15.3	41.7	1.4
**3**	14.0	40.3	0.0

*N*-Cyclo­amino-*o*-sul­fan­il­amides			
**4**	15.2	25.9	3.5
**5**	16.5	22.4	3.4
**6**	30.4	19.6	2.1

**Table 4:** Surface inter­actions of *o*-nitro­sul­fonamides **1**–**3** and *N*-cyclo­amino-*o*-sul­fan­il­amides **4**–**6**

Compound	*d*_norm_		*d*_i_		*d*_e_	
	Minimum value	Maximum value	Minimum value	Maximum value	Minimum value	Maximum value
**1**	−0.2565	0.9743	0.9092	2.3849	0.9083	2.4152
**2**	−0.1071	1.0914	1.0665	2.4390	1.0669	2.4804
**3**	−0.3223	1.7007	0.9319	2.6809	0.9322	2.6406
**4**	−0.3784	1.2864	0.8873	2.4989	0.9319	2.4948
**5**	−0.2060	1.3595	0.9944	2.6968	0.9957	2.5141
**6**	−0.3866	1.2989	0.8842	2.7791	0.8838	2.5779

**Table 5:** Frontier mol­ecular orbital (FMO) energies of synthesized com­pounds **1**–**6**

Parameter (eV)	*o*-Nitrosul­fonamides			*N*-Cyclo­amino-*o*-sul­fan­il­amides		
	**1**	**2**	**3**	**4**	**5**	**6**
HOMO energy (*E*_HOMO_)	−7.228	−7.386	−6.415	−6.189	−6.211	−6.142
LUMO energy (*E*_LUMO_)	−2.982	−2.973	−3.175	−1.627	−1.287	−1.451
Δ*E* gap	4.246	4.413	3.24	4.562	4.924	4.691
Ionization potential (*I*)	7.228	7.386	6.415	6.189	6.211	6.142
Electron affinity (*A*)	2.982	2.973	3.175	1.627	1.287	1.451
Chemical potential (μ)	−5.105	−5.1795	−4.795	−3.908	−3.749	−3.7965
Electronegativity (χ)	5.105	5.1795	4.795	3.908	3.749	3.7965
Global hardness (η)	2.123	2.2065	1.62	2.281	2.462	2.3455
Global softness (*S*)	0.471	0.453	0.617	0.438	0.406	0.426
Global electrophilicity (ω)	27.664	29.597	18.624	17.418	17.301	16.903

**Table 6:** Energies of *o*-nitro­sul­fonamides **1**–**3** and *N*-cyclo­amino-*o*-sul­fan­il­amides **4**–**6** with the hCA II (PDB entry 4iwz) and hCA IX (5fl4) isoenzymes

Entry	Docking score		E-model		Ligand efficiency	
	4iwz	5fl4	4iwz	5fl4	4iwz	5fl4
**1**	−1.351	−0.807	−38.102	−40.980	−0.079	−0.047
**2**	−2.223	−1.977	−41.329	−42.073	−0.124	−0.110
**3**	−1.288	−1.538	−40.486	−38.954	−0.061	−0.073
**4**	−1.645	−1.451	−37.113	−35.898	−0.110	−0.097
**5**	−1.636	−1.605	−41.034	−34.609	−0.102	−0.100
**6**	−0.784	−1.368	−47.945	−42.454	−0.041	0.072
**A**	−2.252	–	−55.202	–	−0.125	–
**B**	–	−1.969	–	−41.029	–	−0.082

Note: **A** is *N*-(5-sulfamoyl-1,3,4-thia­di­azol-2-yl)-2-(thio­phen-2-yl)acetamide and **B** is 5-[1-(naph­tha­len-1-yl)-1,2,3-tri­azol-4-yl]thio­phene-2-sul­fonamide.

**Table 7:** Hydrogen-bond and mixed π-inter­actions (Å) of *o*-nitro­sul­fonamides **1**–**3** and *N*-cyclo­amino-*o*-sul­fan­il­amides **4**–**6** with the hCA II (PDB entry 4iwz) and hCA IX (5fl4) isoenzymes

Isoenzyme/Entry	Carbonic anhydrase II (PDB entry 4iwz)		Carbonic anhydrase IX (PDB entry 5fl4)	
	Hydrogen bond	π–π or π–cation	Hydrogen bond	π–π or π–cation
*o*-Nitro­sul­fonamides				
**1**	GLN_92_ (2.13)		GLN_92_ (2.14)	HID_94_ (4.08) π–cation
**2**	ASN_62_ (2.33), ASN_67_ (2.55)		GLN_71_ (2.28), THR_201_ (2.13)	HID_94_ (4.07) π–cation
**3**	GLN_92_ (2.25), ASN_62_ (2.74)	TRP_5_ (5.36) π–π stacking	GLN_71_ (2.26)	HID_94_ (4.05) π–cation
				
*N*-Cyclo­amino-*o*-sul­fan­il­amides				
**4**	GLN_92_ (2.04), THR_199_ (2.29)		THR_201_ (2.03)	
**5**	GLN_92_ (1.78), THR_199_ (2.26)			
**6**	GLN_92_ (1.88), TRP_5_ (2.11)	THR_199_ (1.81) π–π stacking	GLU92 (2.47)	
**A**	GLN_92_ (2.39), HIE_64_ (2.24)	HIS_94_ (4.75) π–π stacking		
**B**			ASP_13_ (1.59–2.73), VAL_130_ (2.53)	HID_94_ (5.49) π–cation

Note: **A** is *N*-(5-sulfamoyl-1,3,4-thia­di­azol-2-yl)-2-(thio­phen-2-yl)acetamide and **B** is 5-[1-(naph­tha­len-1-yl)-1,2,3-tri­azol-4-yl]thio­phene-2-sul­fonamide.
