# Supplementary material for: N-Cyclo­amino substituent effects on the packing architecture of ortho-sul­fan­il­amide mol­ecular crystals and their in silico carbonic anhydrase II and IX inhibitory activities
Source: Acta Crystallogr C Struct Chem. 2022 Nov 8;78(Pt 12):730–42. doi: 10.1107/S2053229622010130 (PMC9720883; doi:10.1107/S2053229622010130)
Supplement: Supplementary file 14 [file c-78-00730-sup14.pdf]

STRUCTURAL
CHEMISTRY

Volume 78 (2022)

Supporting information for article:

N-Cycloamino substituent effects on the packing architecture of *ortho*-sulfanilamide molecular crystals and their *in silico* carbonic anhydrase II and IX inhibitory activities

Sherif O. Kolade, Josephat U. Izunobi, Allen T. Gordon, Eric C. Hosten, Idris A. Olasupo, Adeniyi S. Ogunlaja, Olayinka T. Asekun and Oluwole B. Familoni

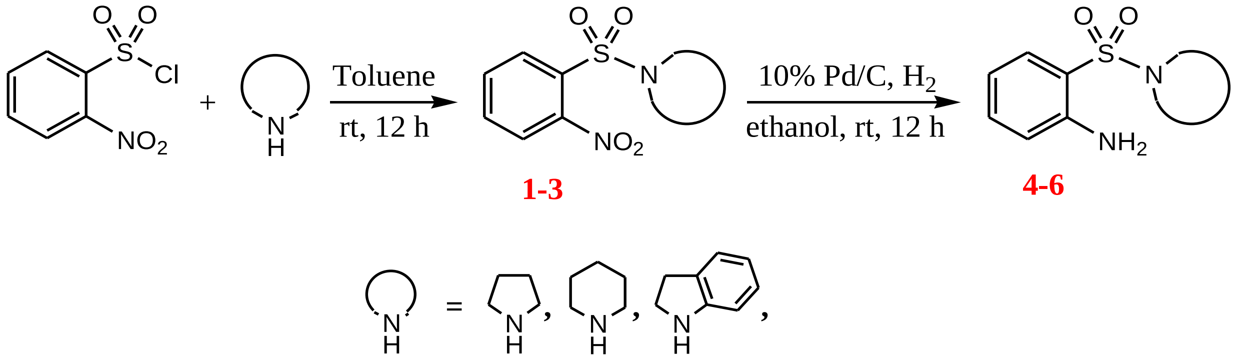

Scheme S1 Synthesis of sulfanilamide from *o*-nitrobenzenesulphonyl chlroide and secondary amines

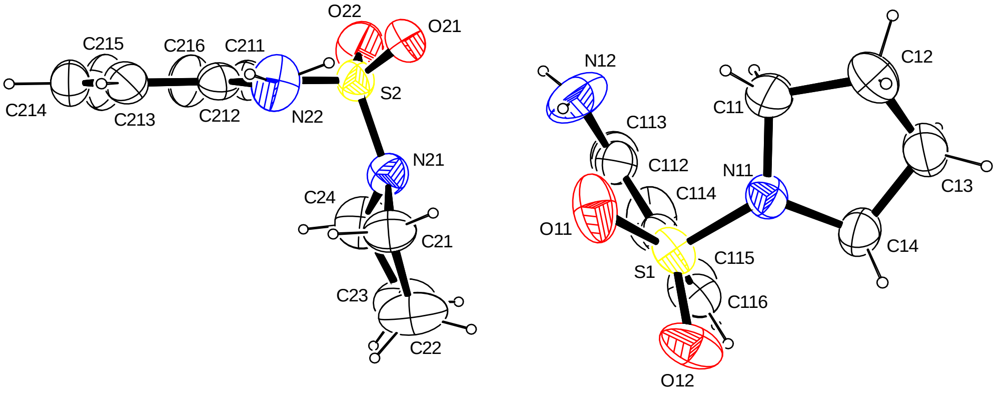

Figure S1: ORTEP diagram of *N*-pyrrolidinyl-*o*-sulfanilamides 4 showing two molecules per asymmetric unit cell. Ellipsoids were drawn at 50% probability level. Minor disorder components are omitted

1

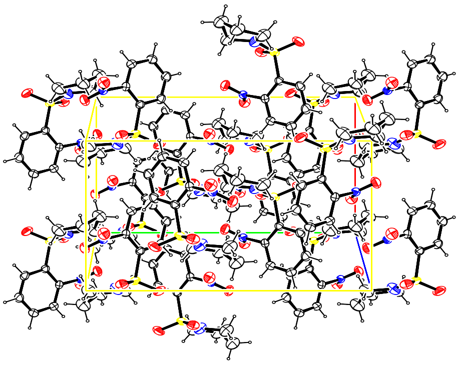

4

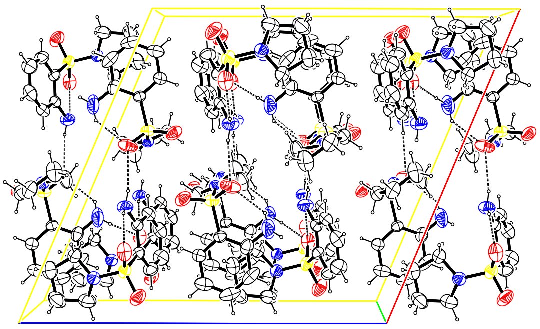

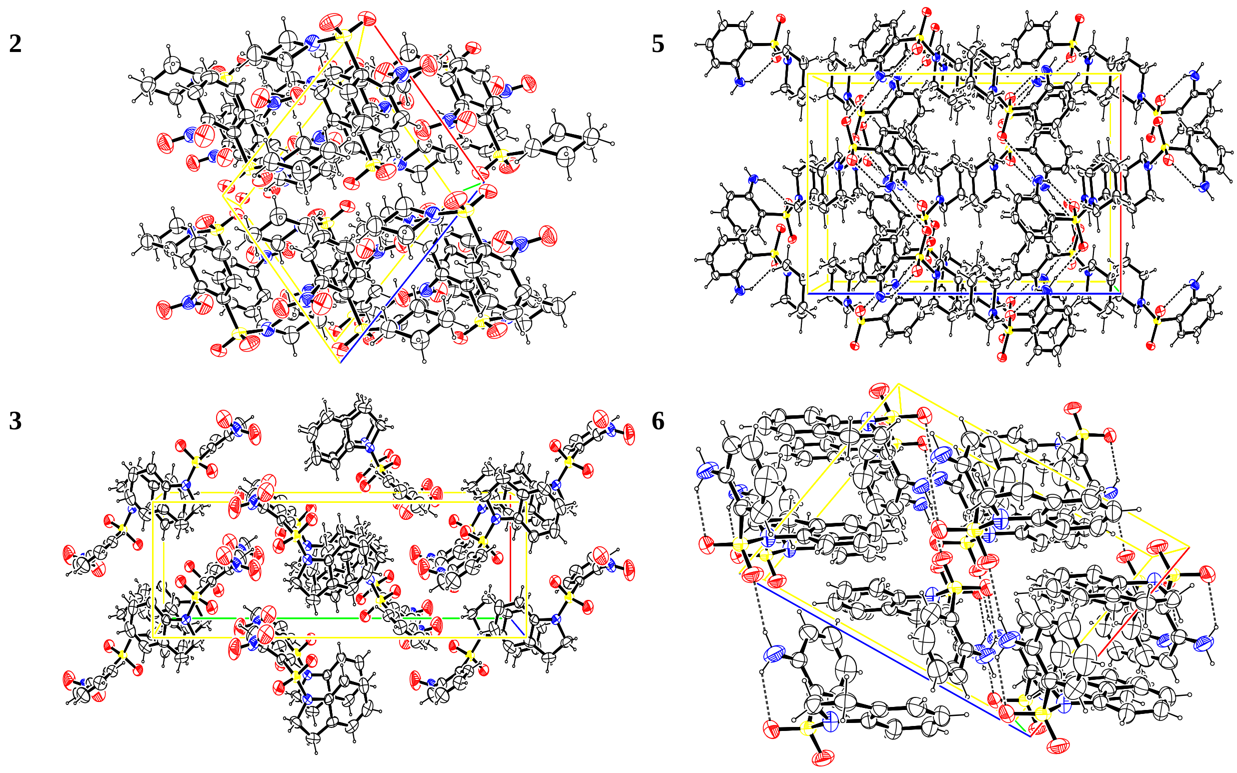

Figure S2: Crystal packing diagrams of compounds **1-6**, drawn normal to (001), (010), (001), (001), (010), (010), (010) and (001), respectively. Ellipsoids were drawn at 50% probability level. Minor disorder components are omitted

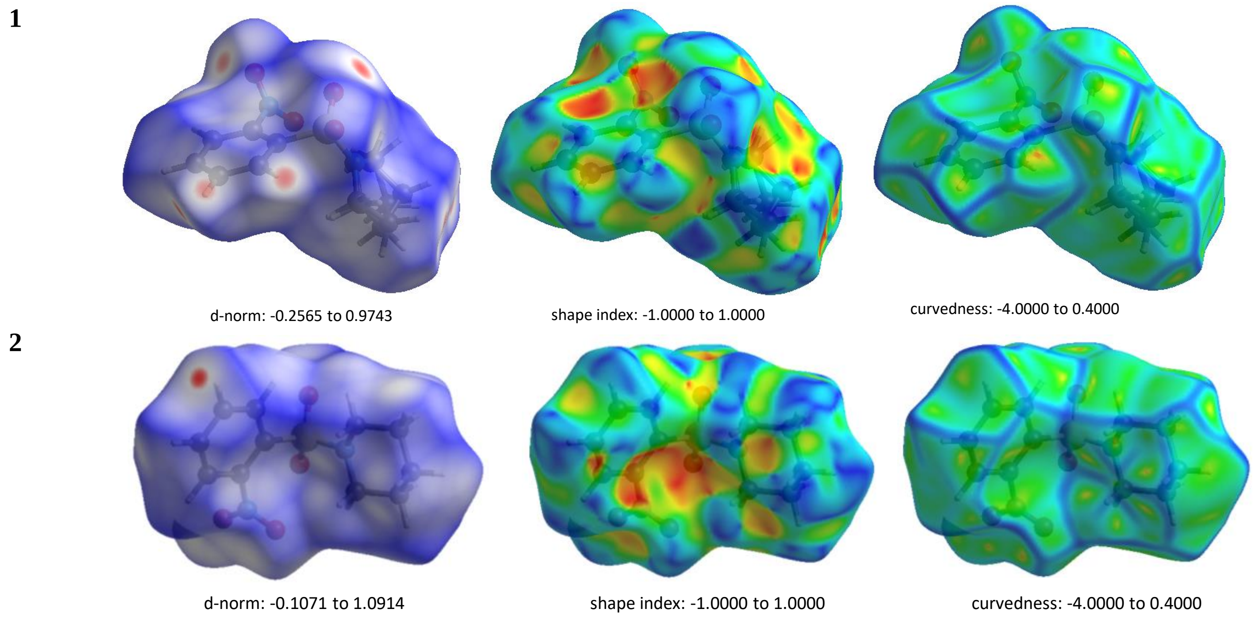

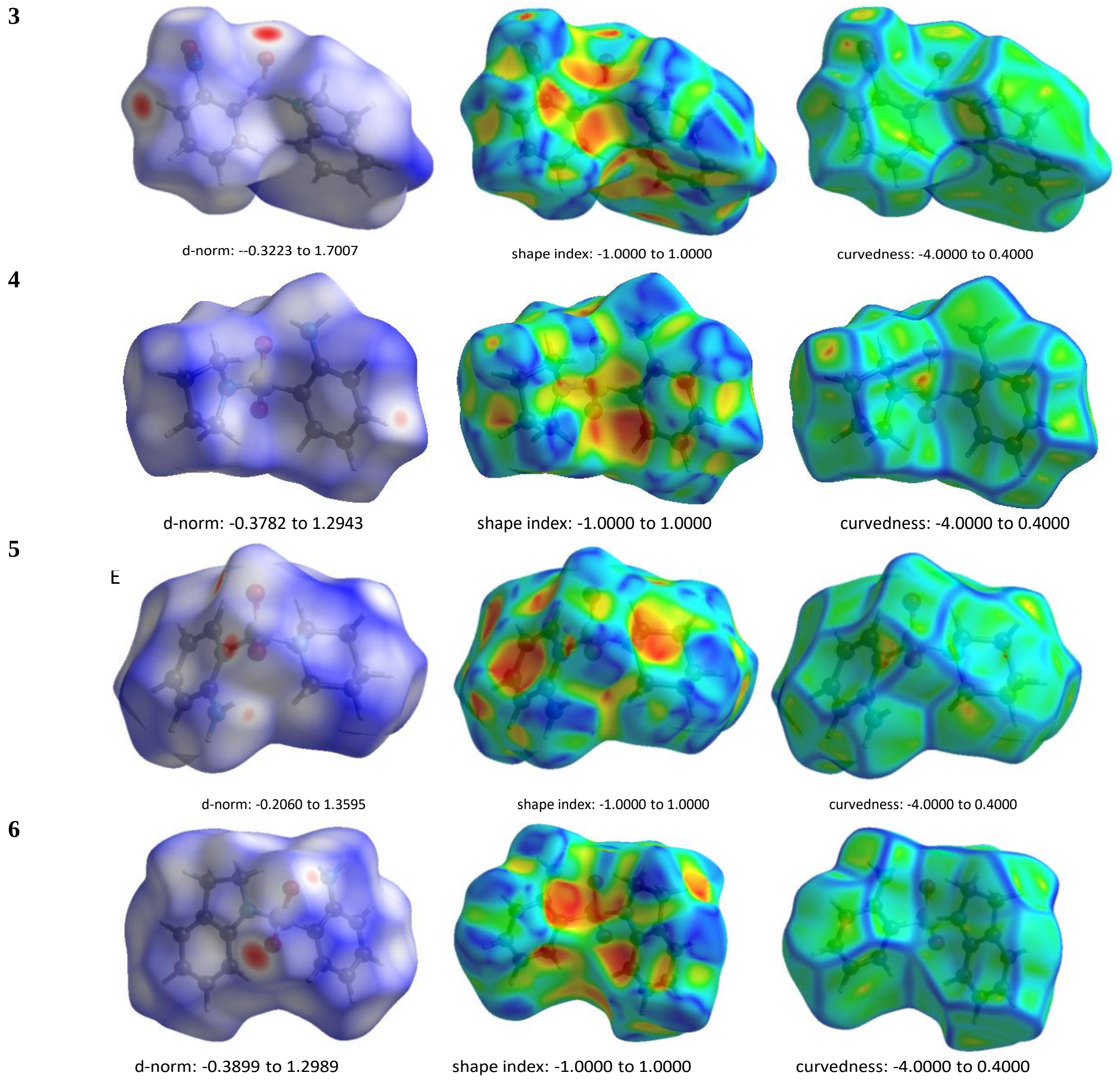

Figure S3: Molecular Hirshfeld surface (d_{norm}, shape index and curvedness) for *o*-nitro sulfonamides (1-3) and *N*-cycloamino-*o*-sulfanilamides (4-6)

Molecules

1

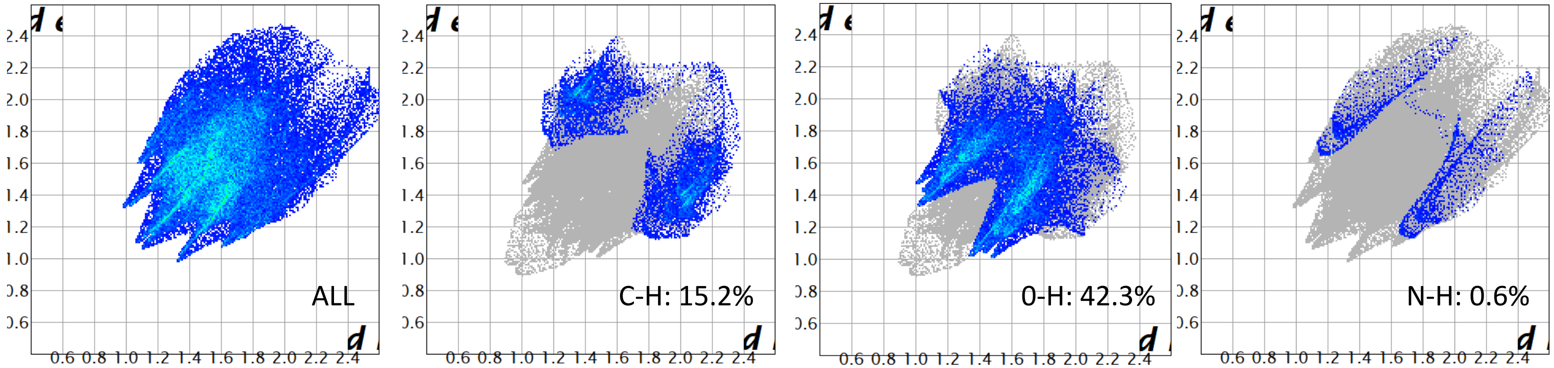

2

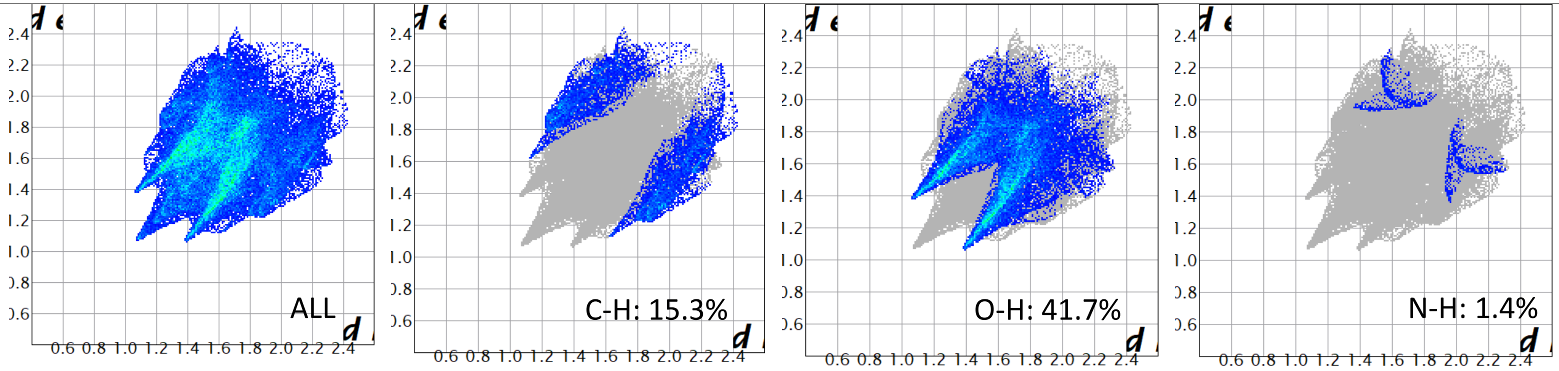

3

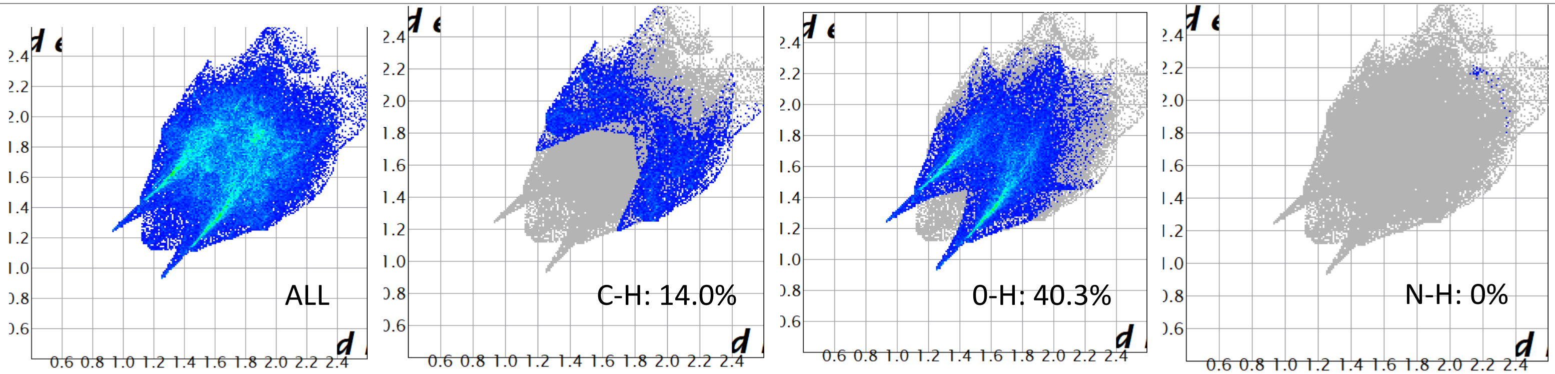

4

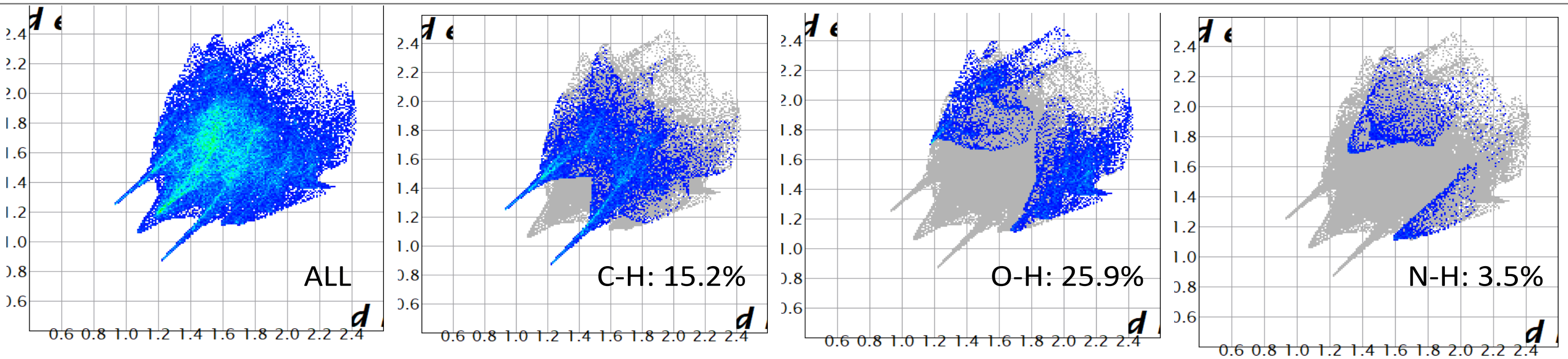

5

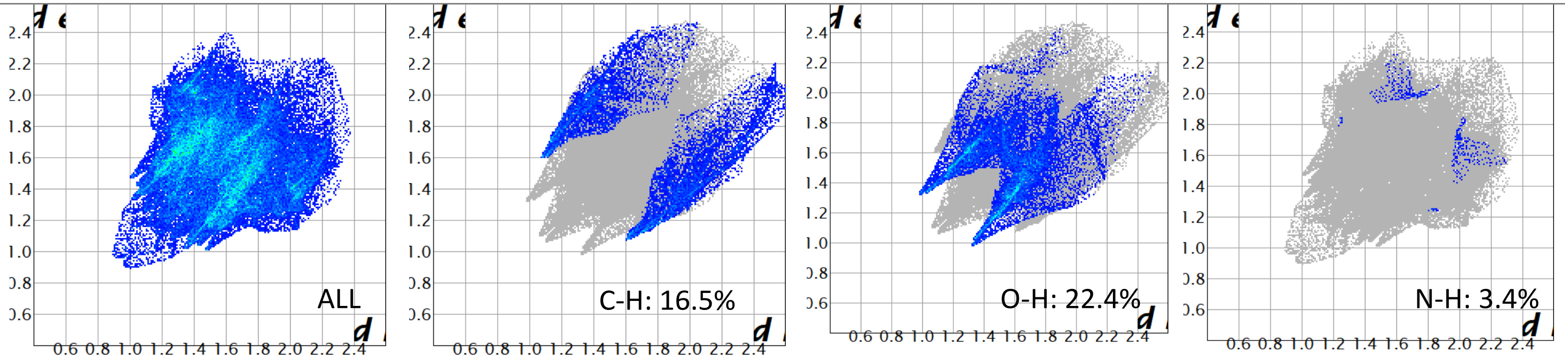

6

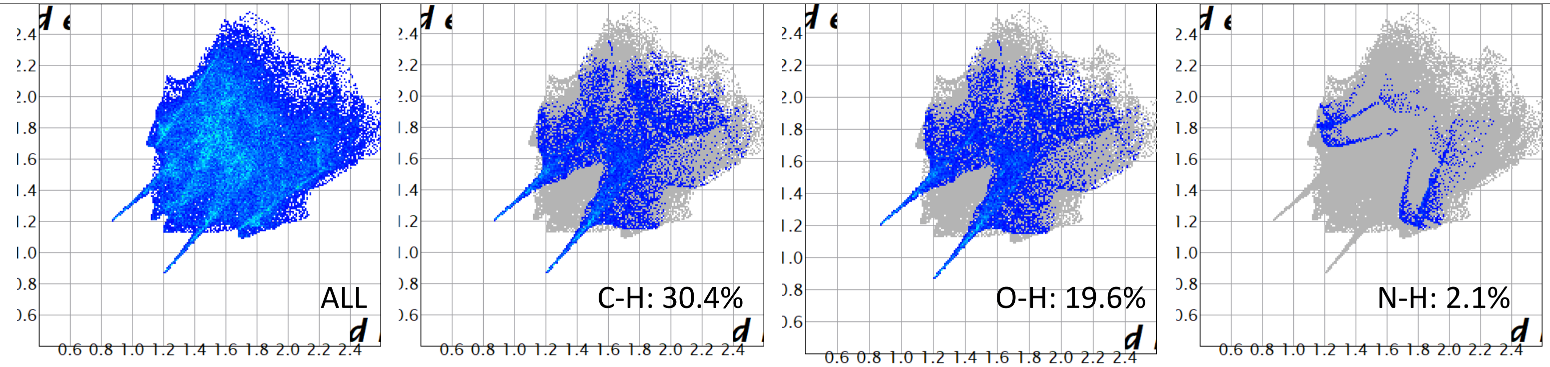

Figure S4: Fingerprint plots of *o*-nitro sulfonamides (1-3) and *N*-cycloamino-*o*-sulfanilamides (4-6), showing full and resolved contacts (into C···H, O···H and N···H), with the percentages of contacts contributed to the total Hirshfeld surface area.

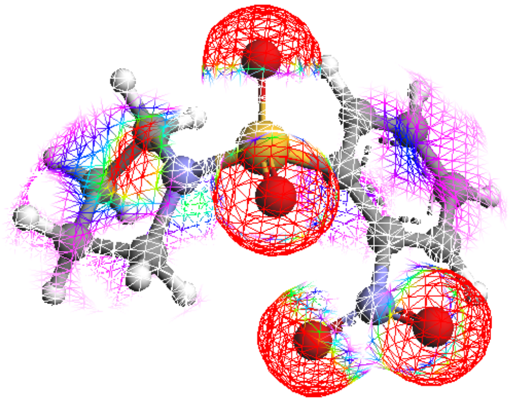

1

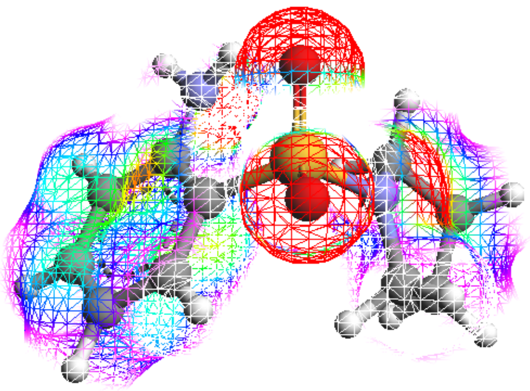

4

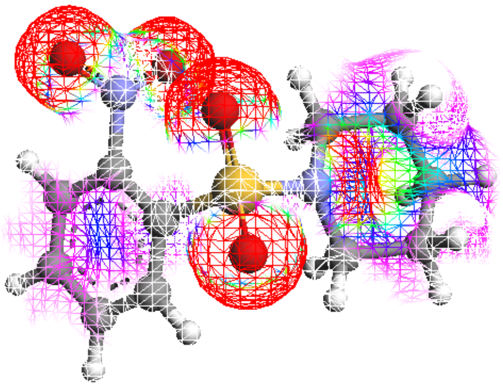

2

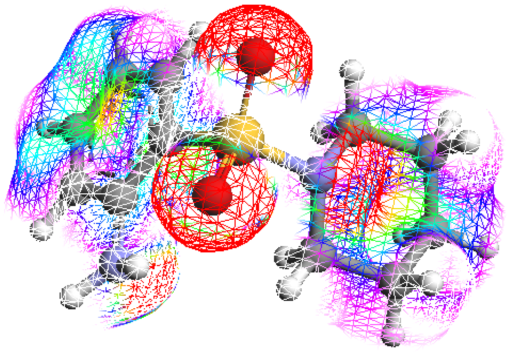

5

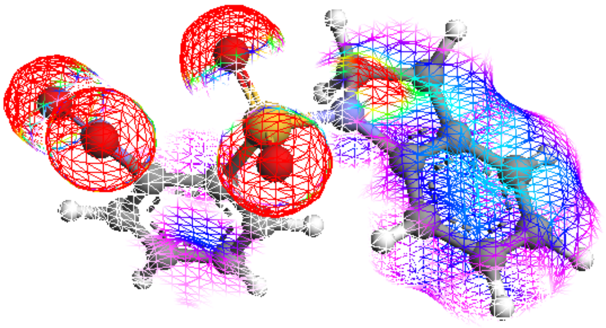

3

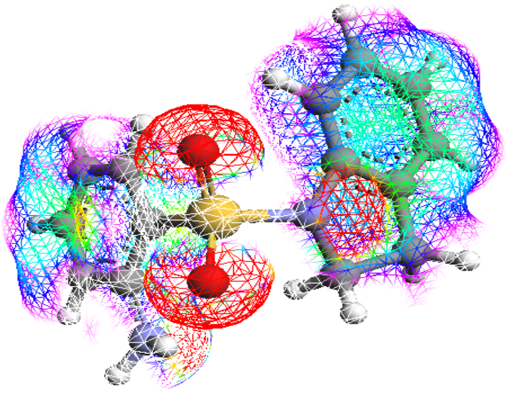

6

Figure S5: Molecular electrostatic potential of **1-6** mapped using 6-311++G** (p,d) level over a range ± 0.03 a.u.

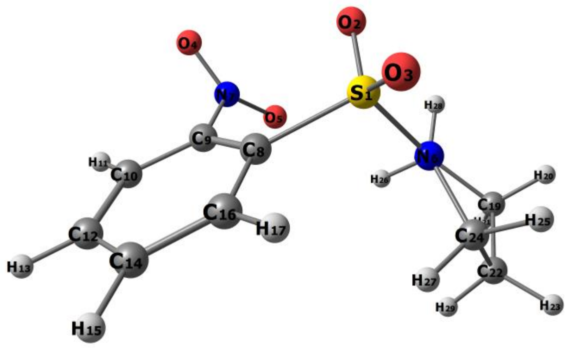

1

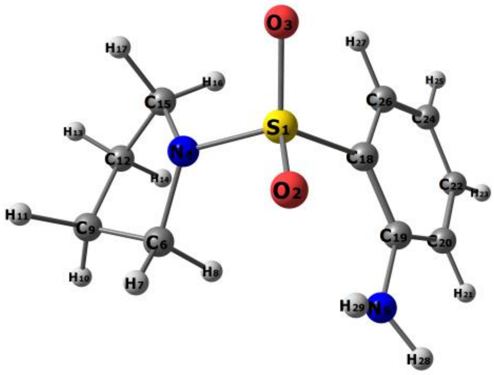

4

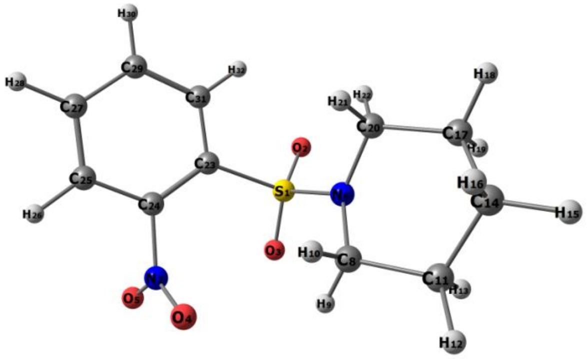

2

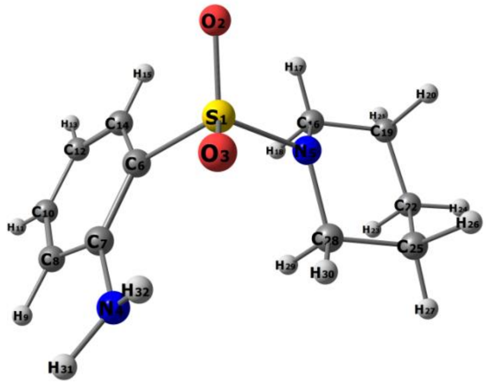

5

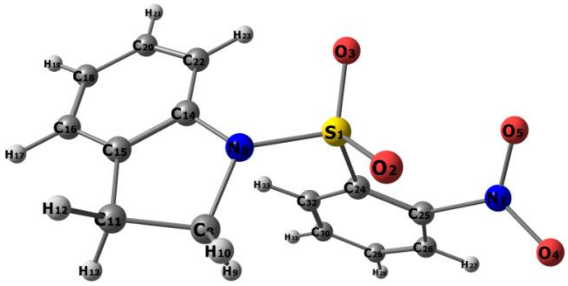

3

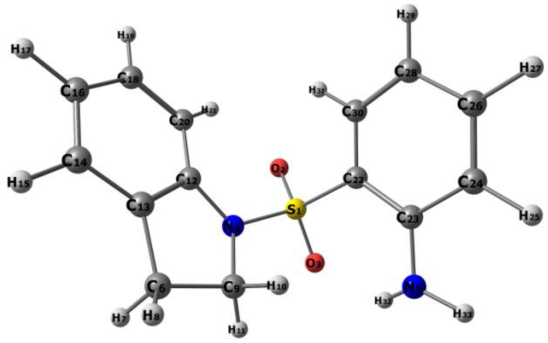

6

Figure S6 Optimized structures of *o*-nitro sulfonamides (**1-3**) and *N*-cycloamino-*o*-sulfanilamides (**4-6**)

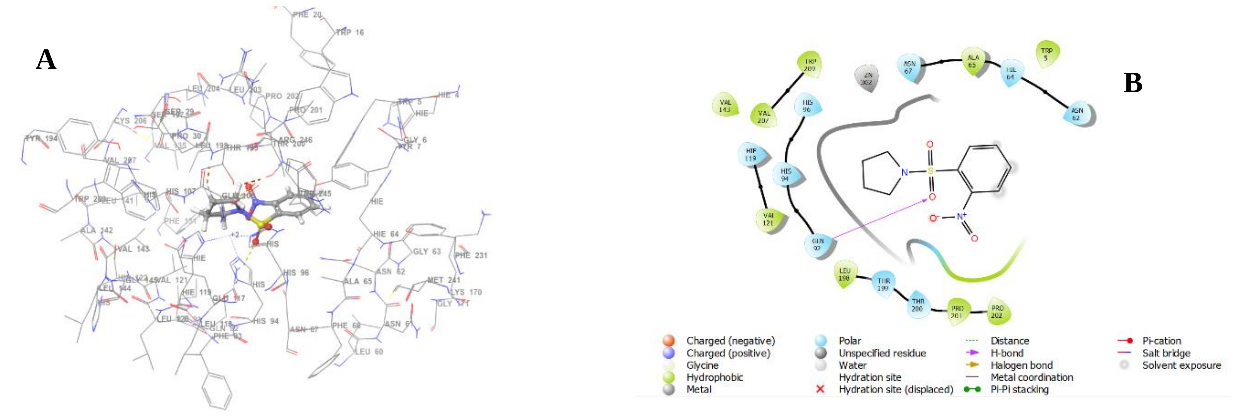

Figure S7 3-D interaction diagram of *o*-nitro sulfonamide **1** and hCA II isoenzyme 4IWZ (**A**) and 2-D interaction diagram depicting *o*-nitro sulfonamide **1** and the binding residues of 4IWZ (**B**)

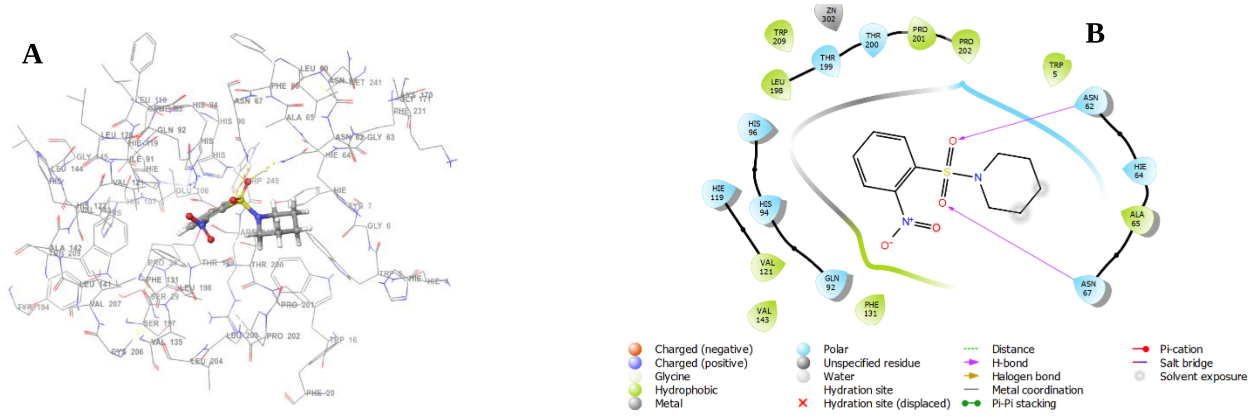

Figure S8 3-D interaction diagram of *o*-nitro sulfonamide **2** and hCA II isoenzyme 4IWZ (**A**) and 2-D interaction diagram depicting *o*-nitro sulfonamide **2** and the binding residues of 4IWZ (**B**)

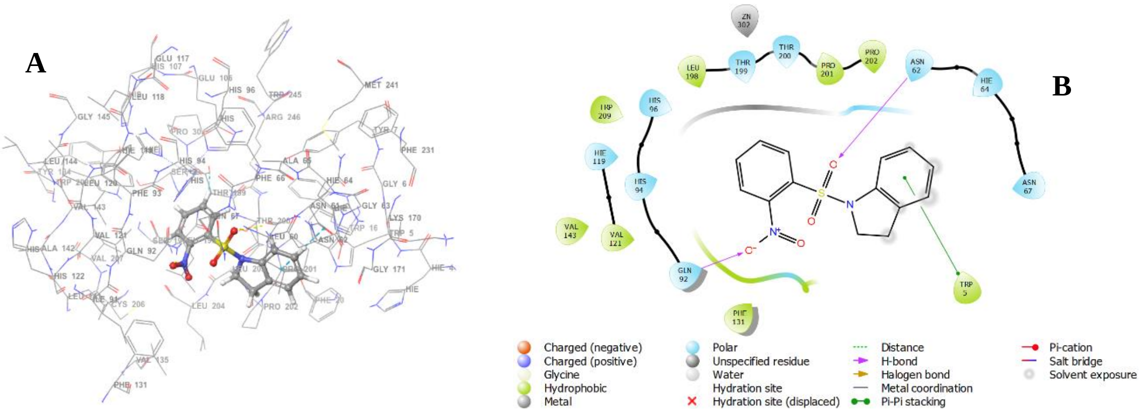

Figure S9 3-D interaction diagram of *o*-nitro sulfonamide **3** and hCA II isoenzyme 4IWZ (**A**) and 2-D interaction diagram depicting *o*-nitro sulfonamide **3** and the binding residues of 4IWZ (**B**)

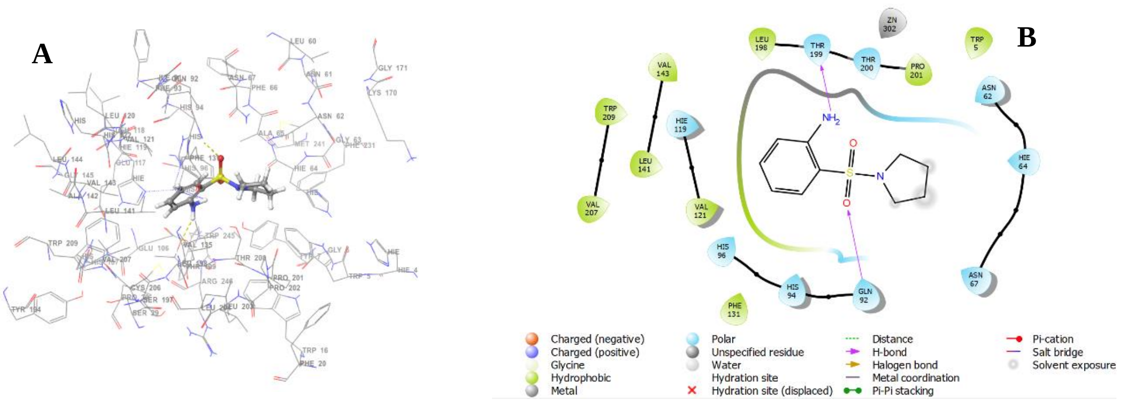

Figure S10 3-D interaction diagram of *N*-cycloamino-*o*-sulfanilamide **4** and hCA II isoenzyme 4IWZ (**A**) and 2-D interaction diagram depicting *N*-cycloamino-*o*-sulfanilamide **4** and the binding residues of 4IWZ (**B**)

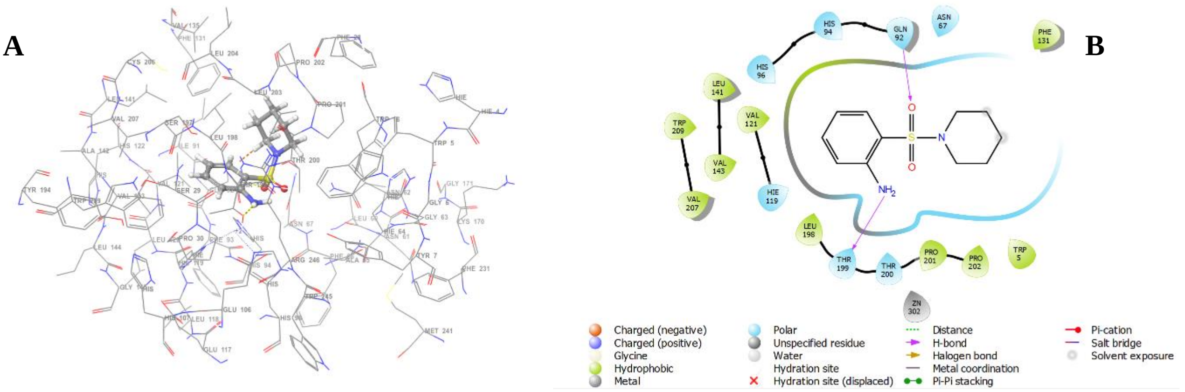

Figure S11 3-D interaction diagram of *N*-cycloamino-*o*-sulfanilamide **5** and hCA II isoenzyme 4IWZ (**A**) and 2-D interaction diagram depicting *N*-cycloamino-*o*-sulfanilamide **5** and the binding residues of 4IWZ (**B**)

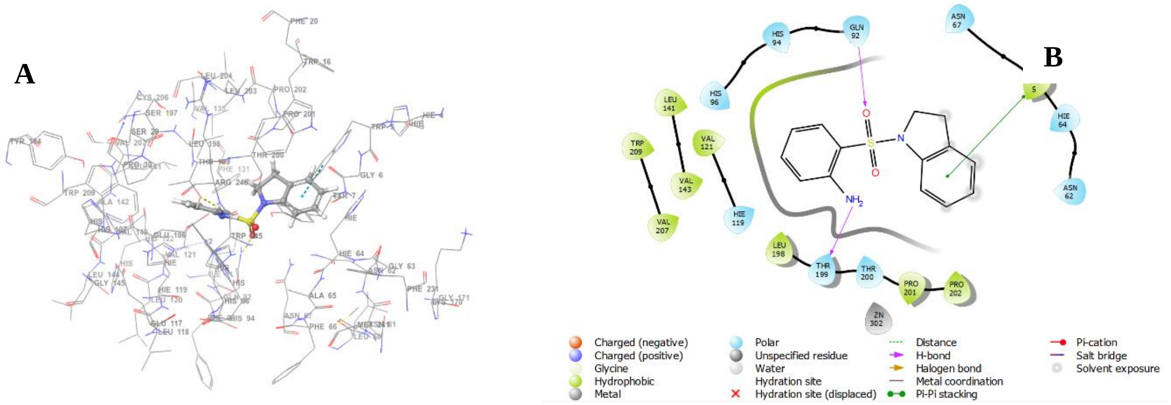

Figure S12 3-D interaction diagram of *N*-cycloamino-*o*-sulfanilamide **6** and hCA II isoenzyme 4IWZ (**A**) and 2-D interaction diagram depicting *N*-cycloamino-*o*-sulfanilamide **6** and the binding residues of 4IWZ (**B**)

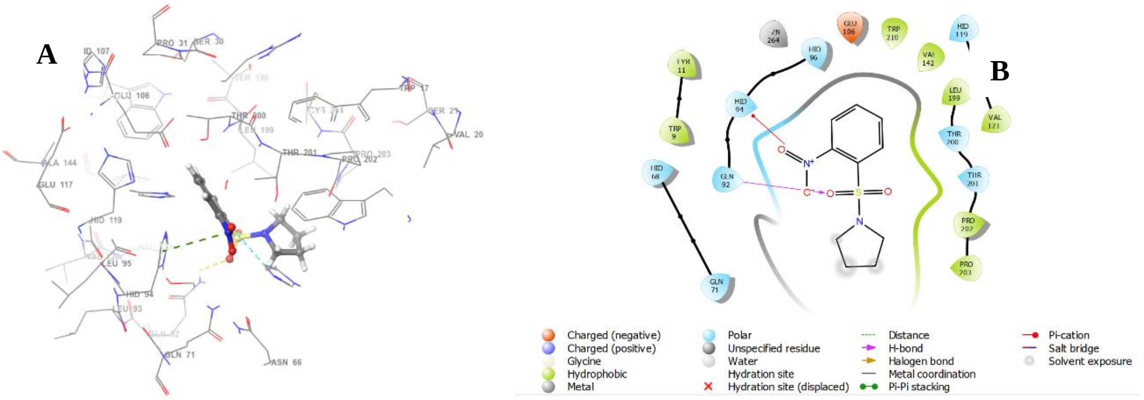

Figure S13 3-D interaction diagram of *o*-nitro sulfonamide **1** and hCA IX isoenzyme 5FL4 (**A**) and 2-D interaction diagram depicting *o*-nitro sulfonamide **1** and the binding residues of 5FL4 (**B**)

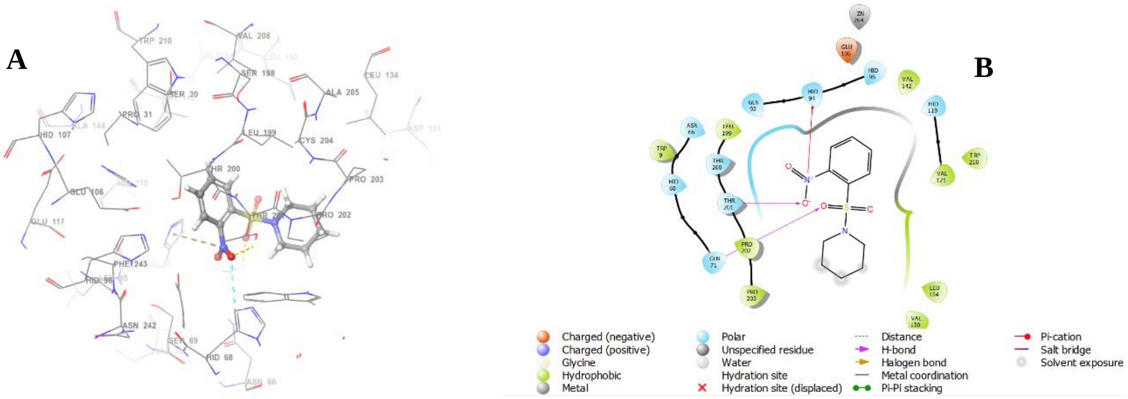

Figure S14 3-D interaction diagram of *o*-nitro sulfonamide **2** and hCA IX isoenzyme 5FL4 (**A**) and 2-D interaction diagram depicting *o*-nitro sulfonamide **2** and the binding residues of 5FL4 (**B**)

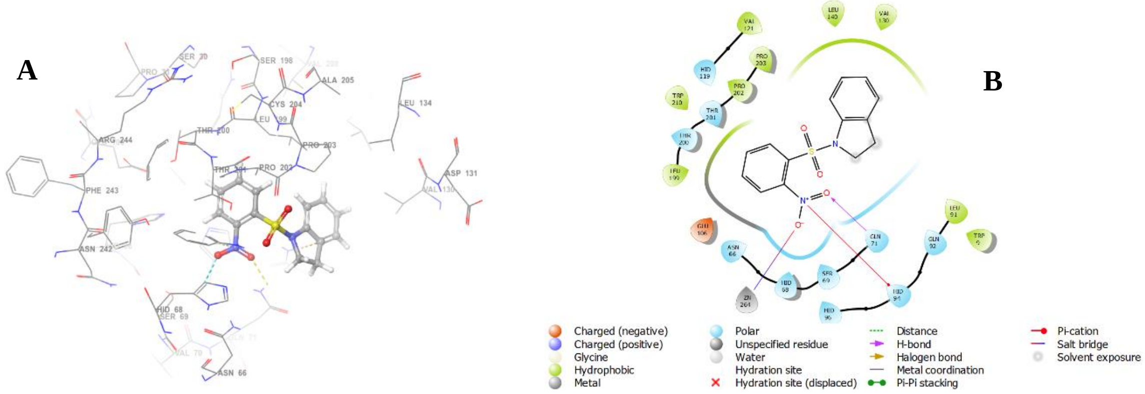

Figure S15 3-D interaction diagram of *o*-nitro sulfonamide **3** and hCA IX isoenzyme 5FL4 (**A**) and 2-D interaction diagram depicting *o*-nitro sulfonamide **3** and the binding residues of 5FL4 (**B**)

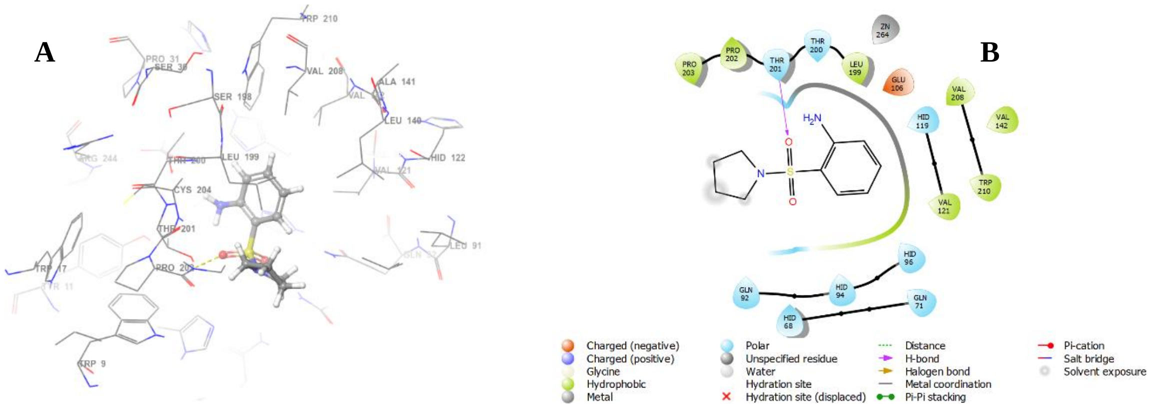

Figure S16 3-D interaction diagram of *N*-cycloamino-*o*-sulfanilamide **4** and hCA IX isoenzyme 5FL4 (**A**) and 2-D interaction diagram depicting *N*-cycloamino-*o*-sulfanilamide **4** and the binding residues of 5FL4 (**B**)

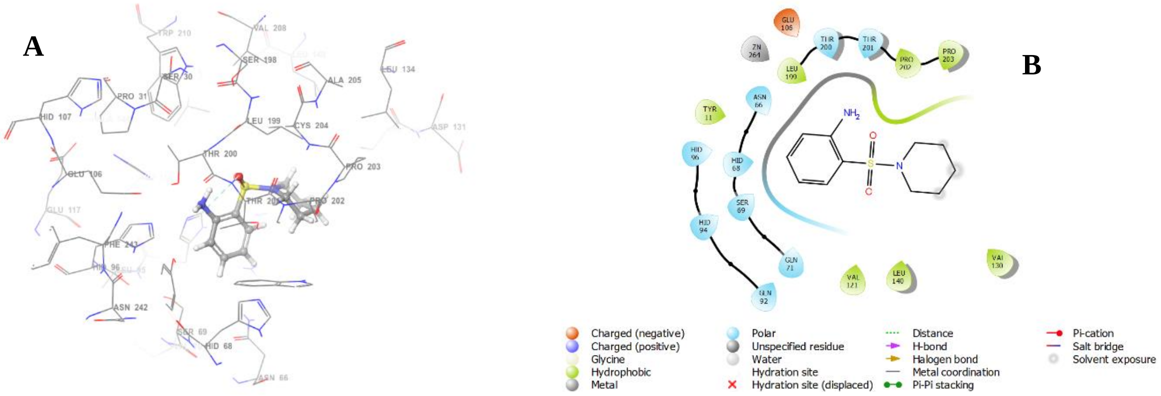

Figure S17 3-D interaction diagram of *N*-cycloamino-*o*-sulfanilamide **5** and hCA IX isoenzyme 5FL4 (**A**) and 2-D interaction diagram depicting *N*-cycloamino-*o*-sulfanilamide **5** and the binding residues of 5FL4 (**B**)

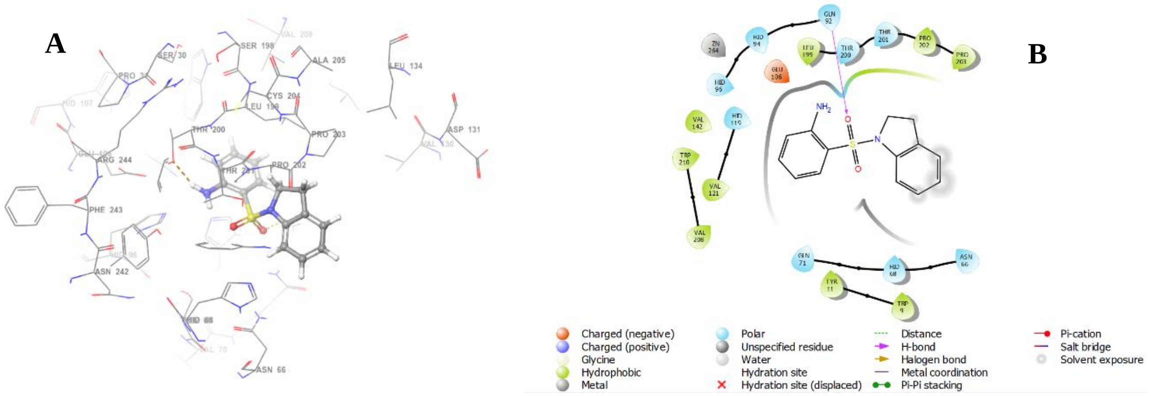

Figure S18 3-D interaction diagram of *N*-cycloamino-*o*-sulfanilamide **6** and hCA IX isoenzyme 5FL4 (**A**) and 2-D interaction diagram depicting *N*-cycloamino-*o*-sulfanilamide **6** and the binding residues of 5FL4 (**B**)

Table S1: Selected bond lengths (Å) and angles (°) of, *o*-nitro sulfonamides (1-3) and *N*-cycloamino-*o*-sulfanilamides (4-6)

***o*-Nitro sulfonamides**					***N*-Cycloamino-*o*-sulfanilamides**				
**1**	S1–O1	1.4285 (13)	O1–S1–O2	119.93 (9)	**4**	S1–O11	1.4369 (14)	O11–S1–O12	118.78 (8)
	S1–O2	1.4300 (16)	N1–S1–C21	107.89 (8)		S1–O12	1.4237 (13)	N11–S1–C111	109.00 (7)
	S1–N1	1.5911 (17)	C11–N1–C14	108.3 (4)		S1–N11	1.6127 (13)	C11–N11–C14	110.00 (12)
	S1–C21	1.7818 (17)	C15–N1–C18	117.7 (5)		S1–C111	1.7496 (16)	C111–C112–N12	123.25 (18)
	C22–N2	1.4707 (19)	C21–C22–N2	121.65 (14)		C112–N12	1.365 (3)	O21–S2–O22	118.33 (8)
						S2–O21	1.4331 (14)	N21–S2–C211	108.86 (7)
						S2–O22	1.4252 (14)	C21–N21–C24	107.30 (15)
**2**	S1–O1	1.4253 (13)	O1–S1–O2	119.57 (8)		S2–N21	1.6159 (15)	C211–C212–N22	116.99 (14)
	S1–O2	1.4263 (11)	N1–S1–C21	108.60 (7)		S2–C211	1.7560 (15)	O11–S1–O12	118.78 (8)
	S1–N1	1.6159 (13)	C11–N1–C15	114.07 (13)		C212–N22	1.372 (2)	N11–S1–C111	109.00 (7)
	S1–C21	1.7886 (15)	C21–C22–N2	122.27 (13)					
	C22–N2	1.4713 (19)			**5**	S1–O1	1.4383 (8)	O1–S1–O2	118.52 (5)
						S1–O2	1.4369 (9)	N2–S1–C11	107.84 (5)
						S1–C11	1.7612 (13)	C11–C12–N1	124.15 (11)
**3**	S1–O1	1.4237 (13)	O1–S1–O2	120.12 (7)		S1–N2	1.6380 (10)	C21–N2–C25	112.41 (9)
	S1–O2	1.4190 (13)	N1–S1–C21	106.04 (7)		C12–N1	1.3651 (17)		
	S1–N1	1.6022 (14)	C1–N1–C11	109.98 (13)					
	S1–C21	1.7821 (15)	C21–C22–N2	121.43 (14)	**6**	S1–O1	1.4277 (13)	O1–S1–O2	119.06 (7)
	N1–C1	1.485 (2)				S1–O2	1.4317 (13)	N1–S1–C21	108.24 (7)
	N1–C11	1.428 (2)				S1–N1	1.6205 (14)	C2–N1–C11	109.39 (13)
	C22–N2	1.478 (2)				S1–C21	1.7486 (16)	C21–C22–N2	123.82 (15)
						N1–C2	1.482 (2)		
						N1–C11	1.420 (2)		
						C22–N2	1.358 (2)		

FTIR, NMR and MS Spectra of *N*-Cycloamino-*o*-nitrobenzenesulphonamides (1-3) and *N*-Cycloamino-*o*-sulphanilamides (4-6)

***N*-Cycloamino-*o*-nitrobenzenesulphonamides**

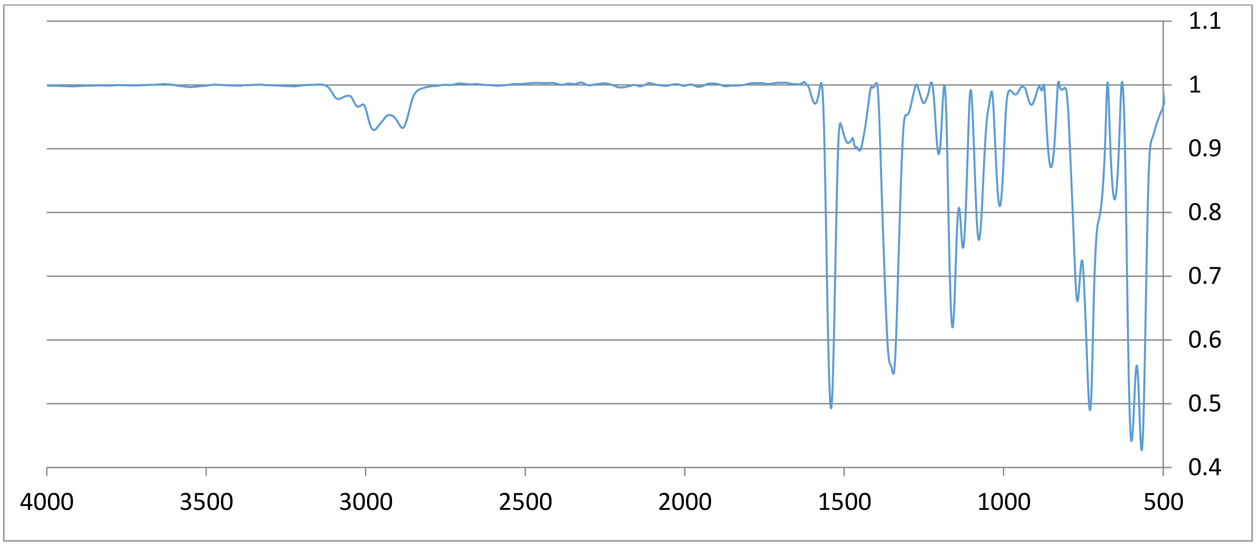

Figure S19: Infrared spectrum of *N*-Pyrrolidinyl-*o*-nitrobenzenesulphonamide (1)

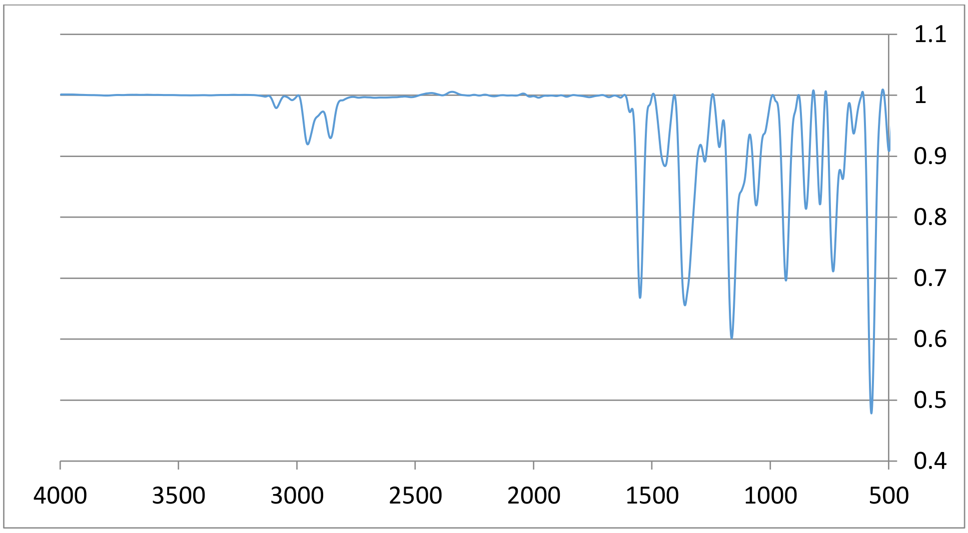

Figure S20: Infrared spectrum of *N*-Piperidinyl-*o*-nitrobenzenesulphonamide (2)

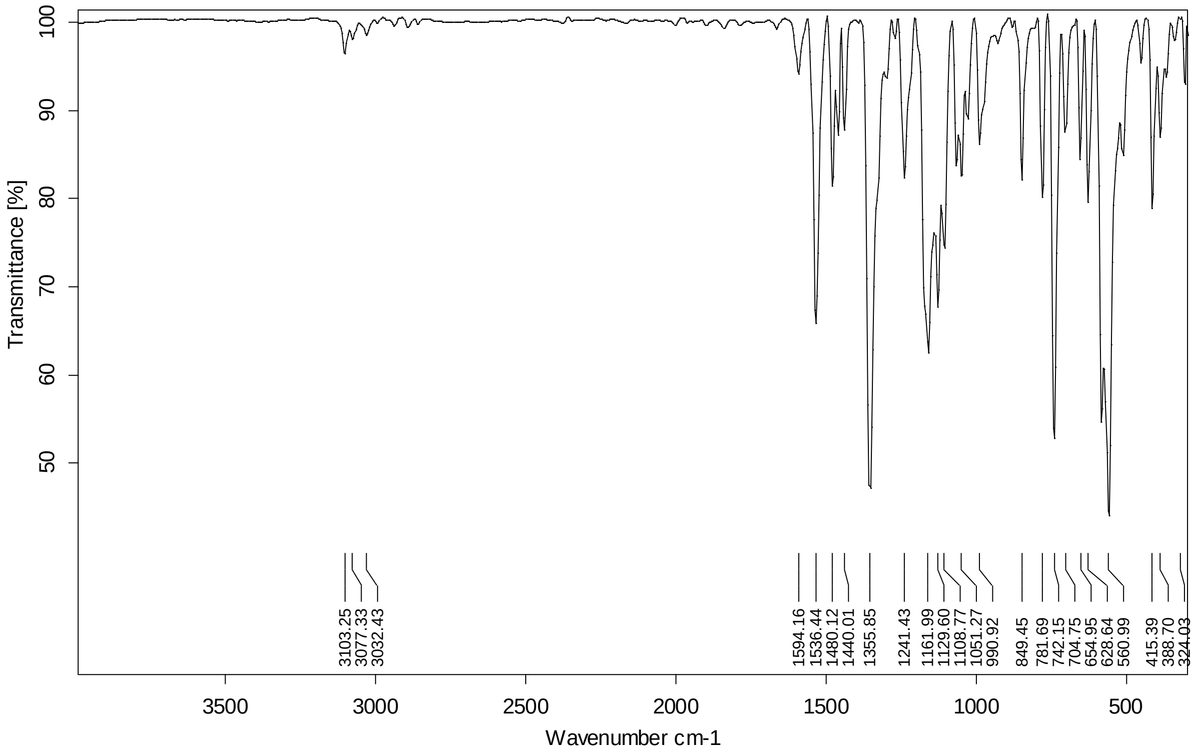

C:\NMMU\SHERIF\N-(2-Nitrobenzenesulphonyl)Indoline.0	N-(2-Nitrobenzenesulphonyl)Indoline	Yellow crystals	08/10/2019

Page 1/1

Figure S21: Infrared spectrum of *N*-Indolinyl-*o*-nitrobenzenesulphonamide (3)

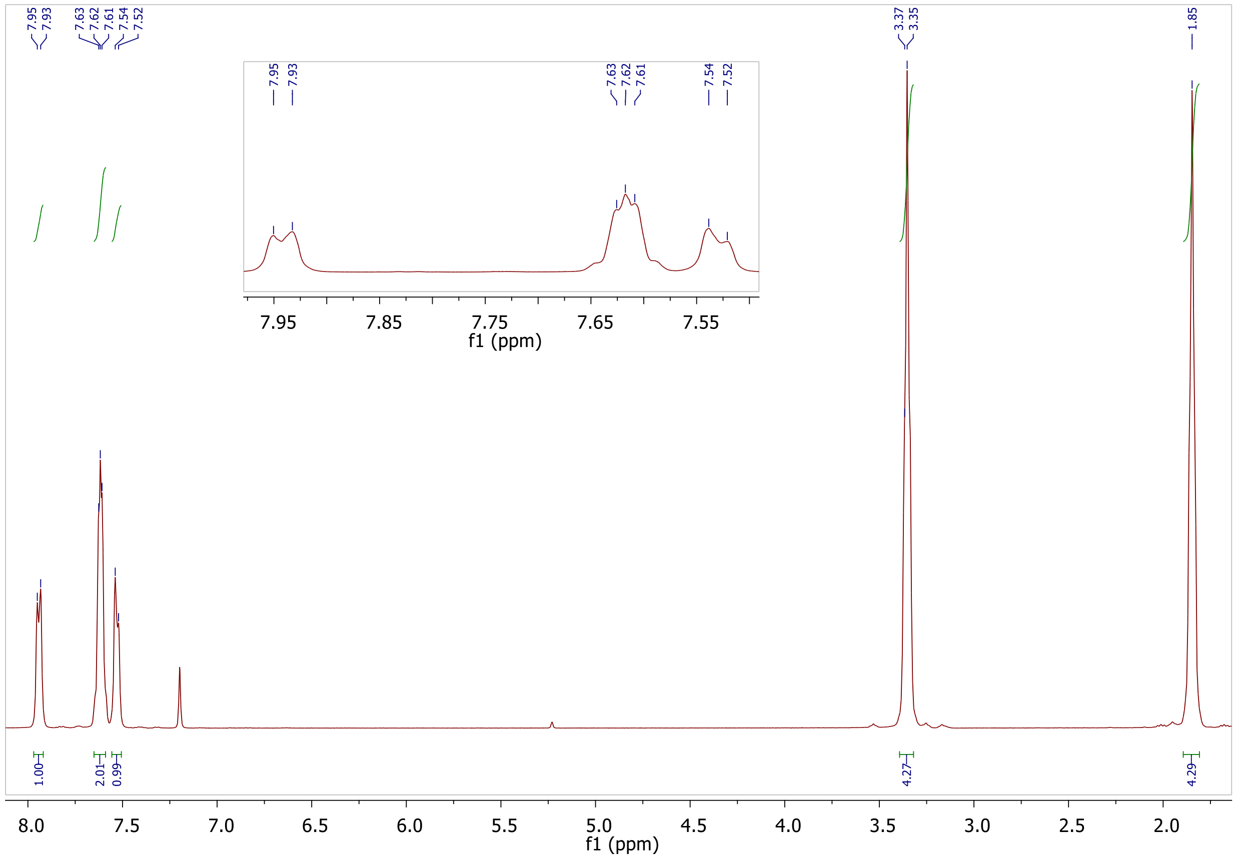

Figure S22: ^{1}H-NMR spectrum of *N*-Pyrrolidinyl-*o*-nitrobenzenesulphonamide (1)

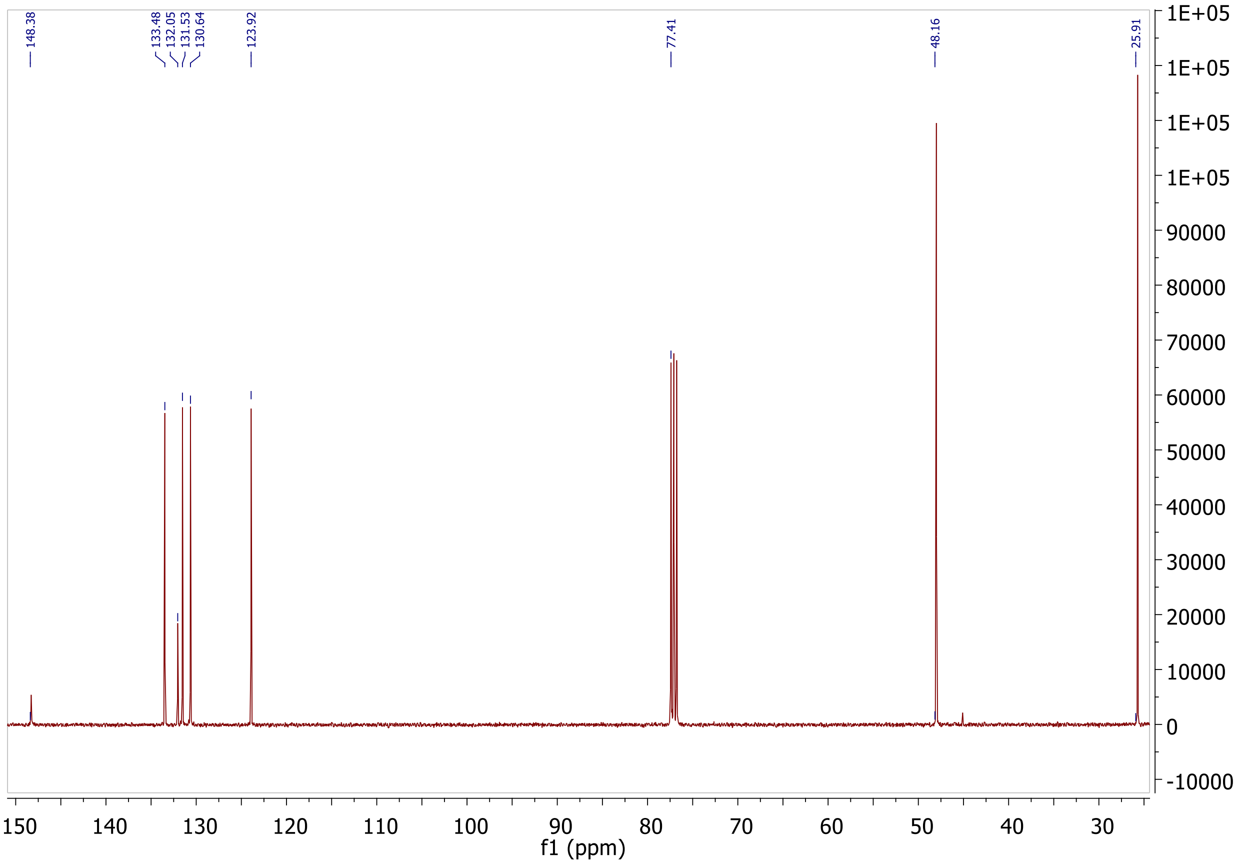

Figure S23: ^{13}C-NMR spectrum of *N*-Pyrrolidinyl-*o*-nitrobenzenesulphonamide (1)

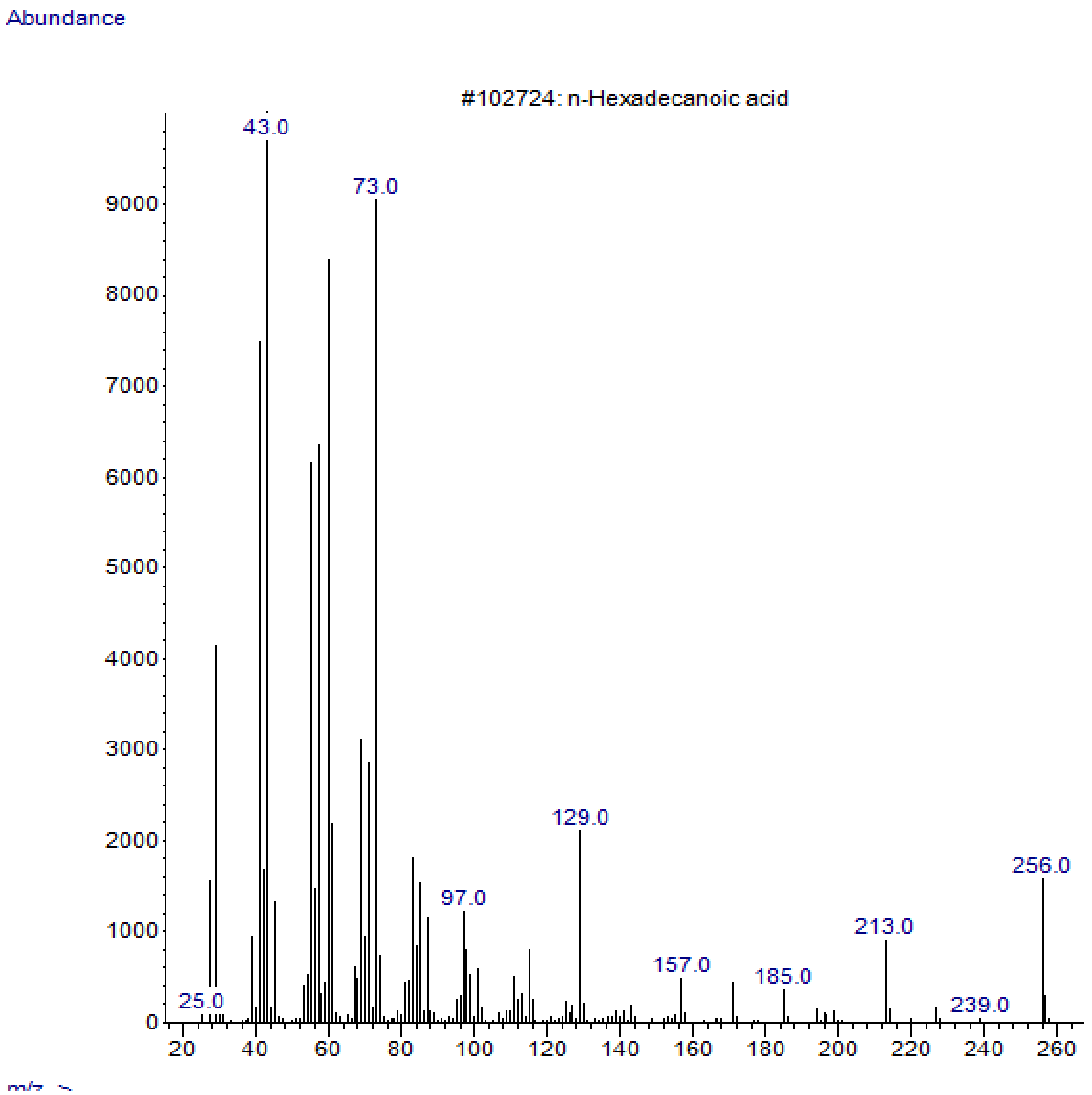

Figure S24: MS spectrum of *N*-Pyrrolidinyl-*o*-nitrobenzenesulphonamide (1)

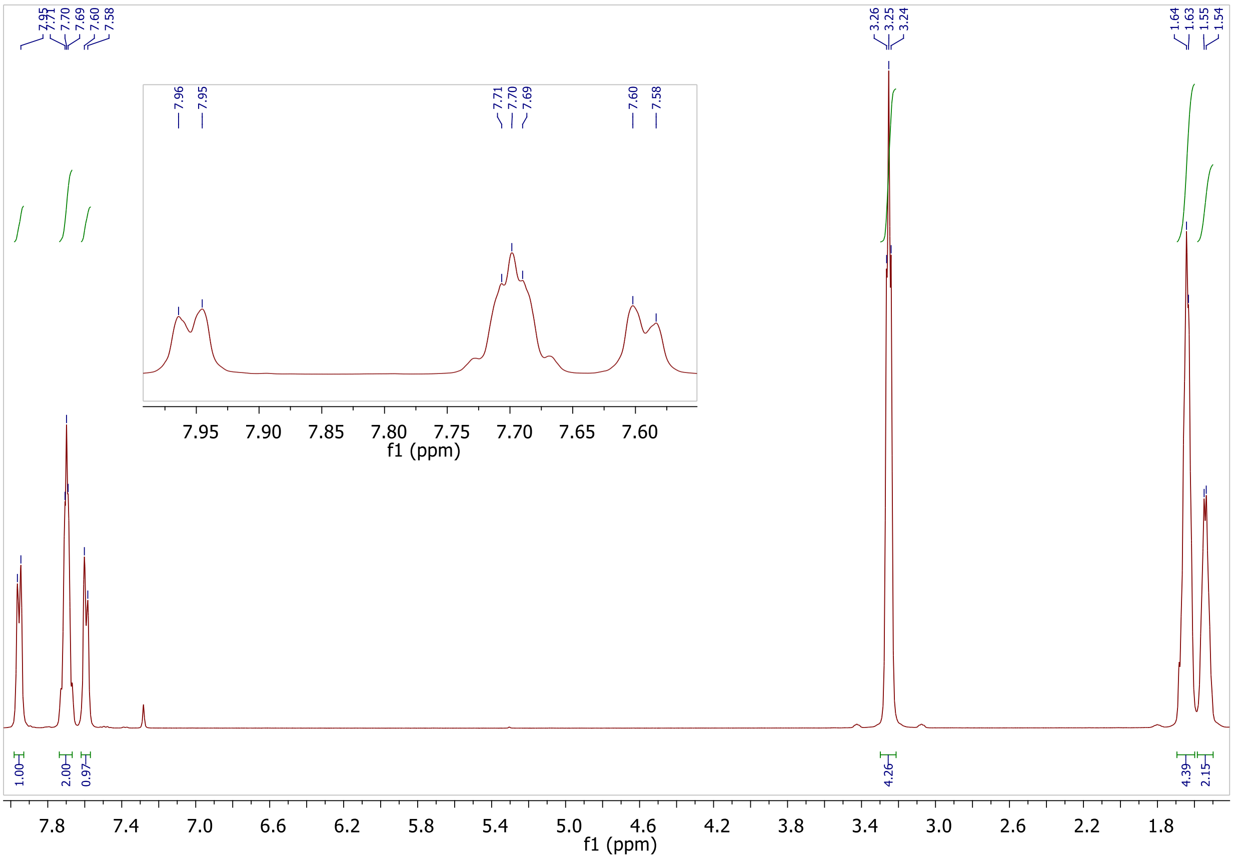

Figure S25: ^{1}H-NMR spectrum of *N*-Piperidinyl-*o*-nitrobenzenesulphonamide (**2**)

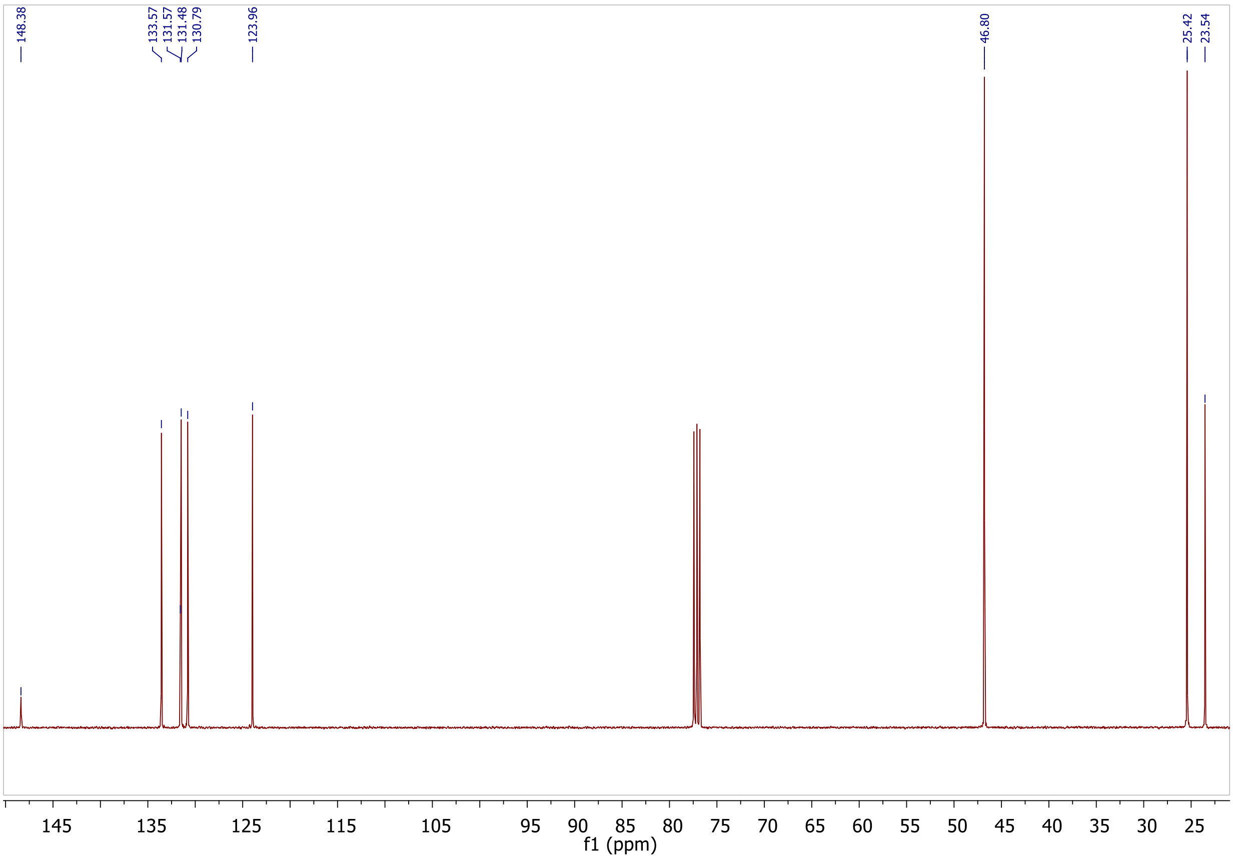

Figure S26: ^{13}C-NMR spectrum of *N*-Piperidinyl-*o*-nitrobenzenesulphonamide (**2**)

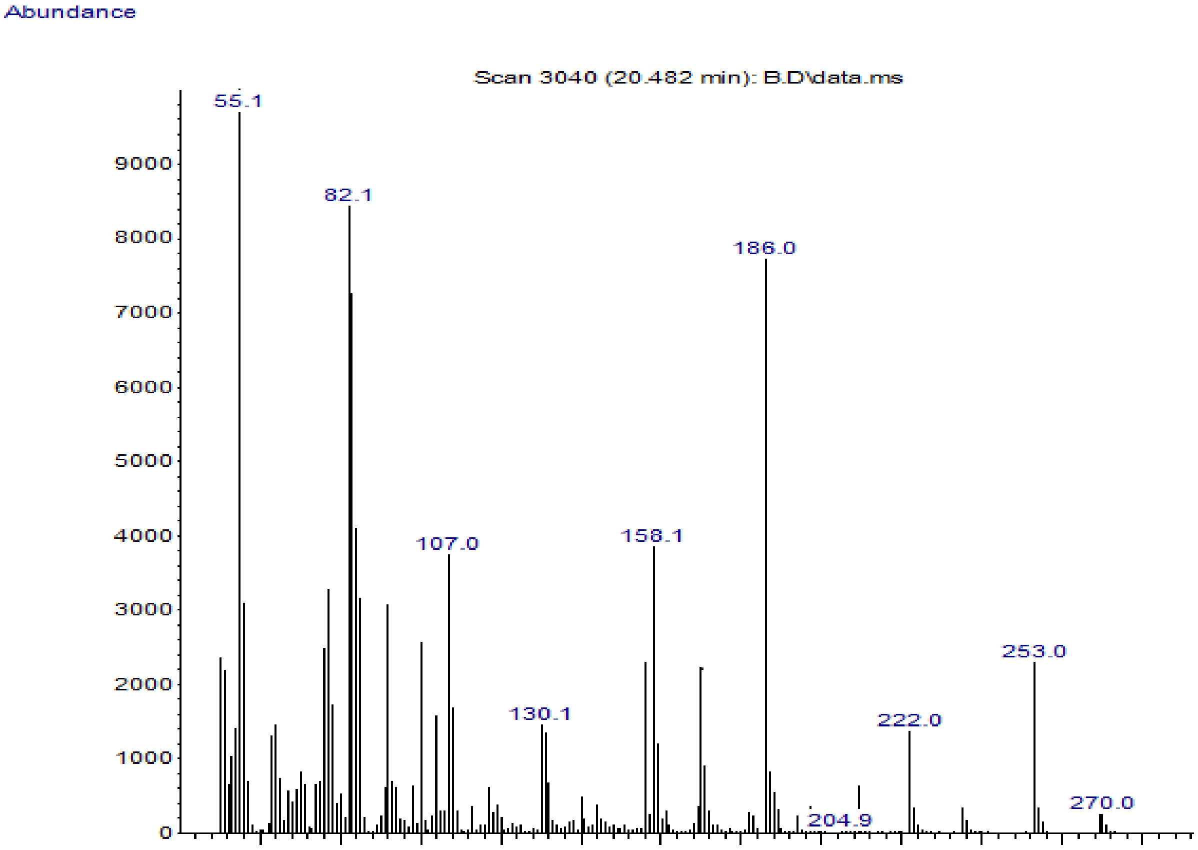

Figure S27: MS spectrum of *N*-Piperidinyl-*o*-nitrobenzenesulphonamide (**2**)

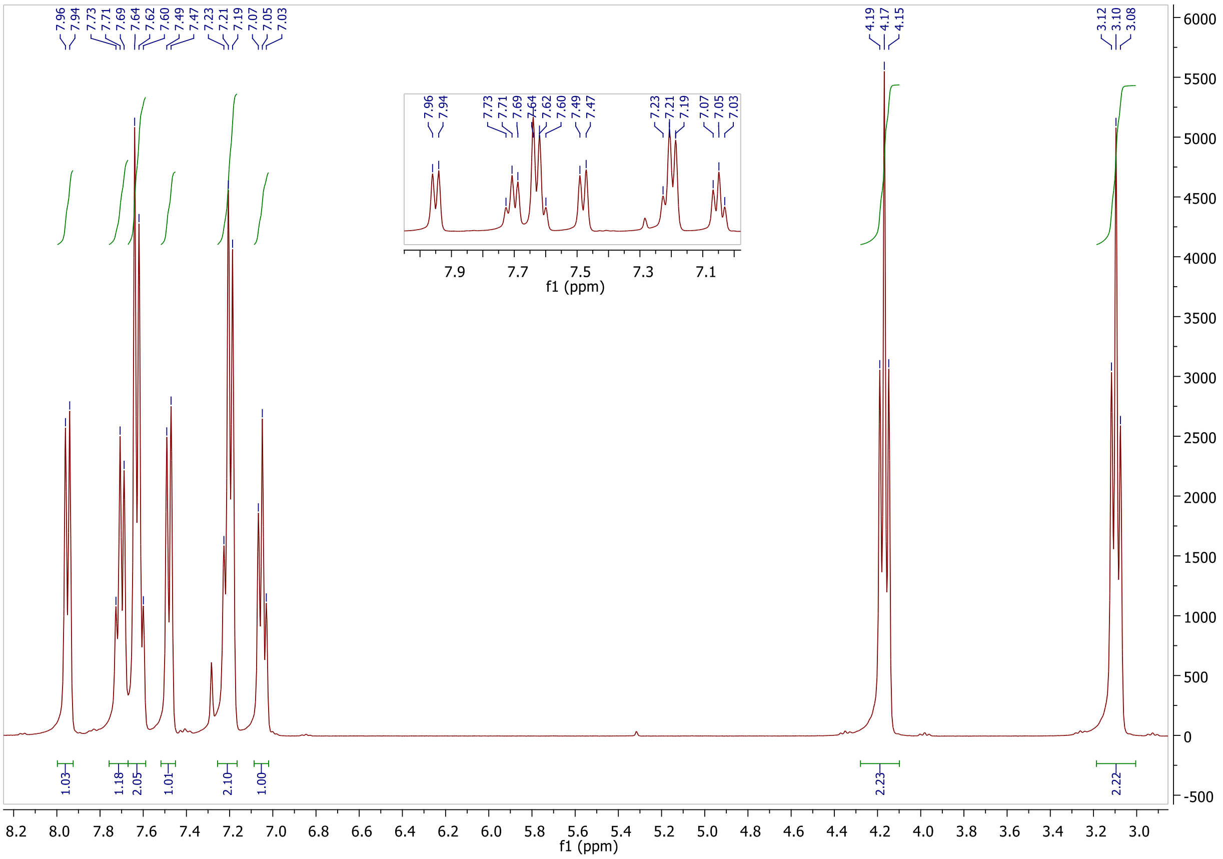

Figure S28: ^{1}H-NMR spectrum of *N*-Indolinyl-*o*-nitrobenzenesulphonamide (**3**)

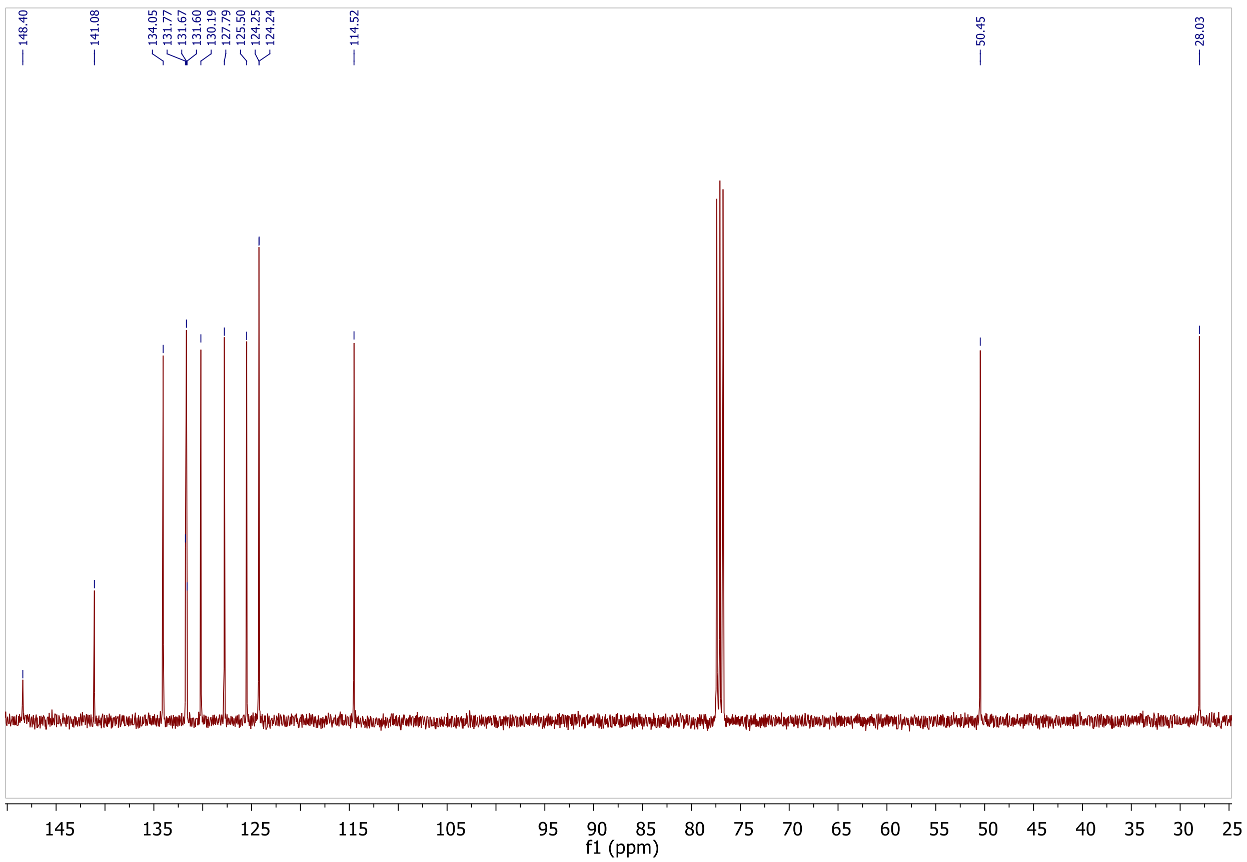

Figure S29: ^{13}C-NMR spectrum of *N*-Indolinyl-*o*-nitrobenzenesulphonamide (**3**)

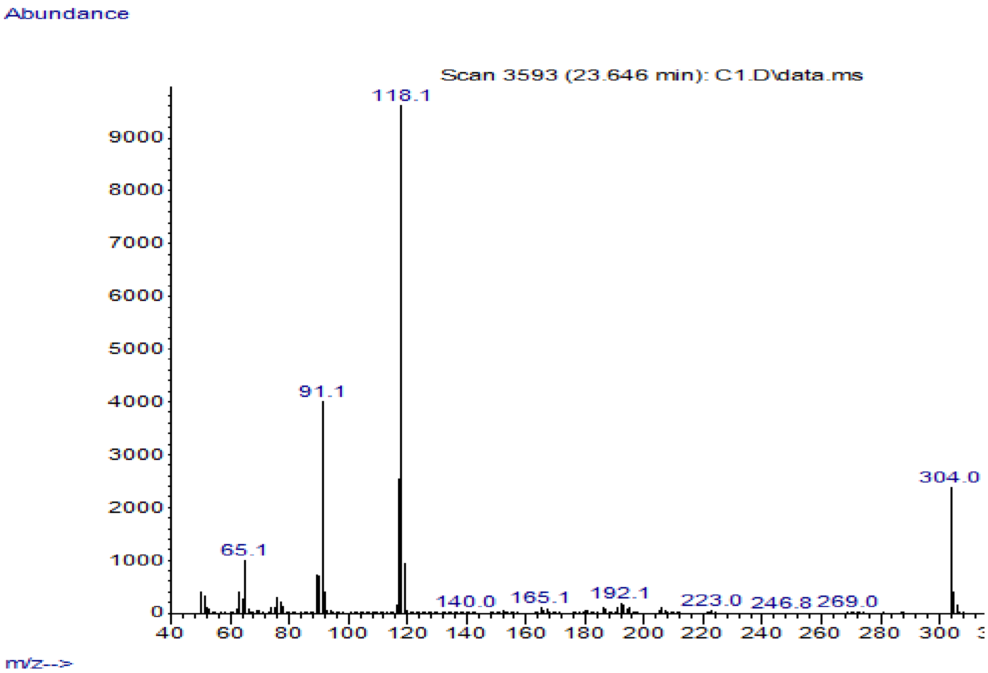

Figure S30: MS spectrum of *N*-Indolinyl-*o*-nitrobenzenesulphonamide (**3**)

N-Cycloamino-*o*-sulphanilamides

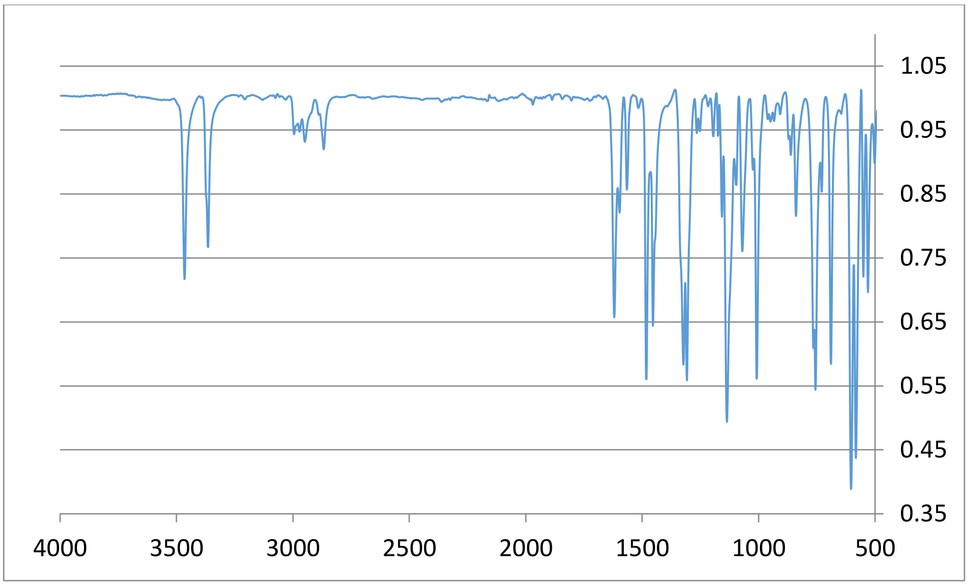

Figure S31: Infrared spectrum of *N*-Pyrrolidinyl-*o*-sulphanilamide (**4**)

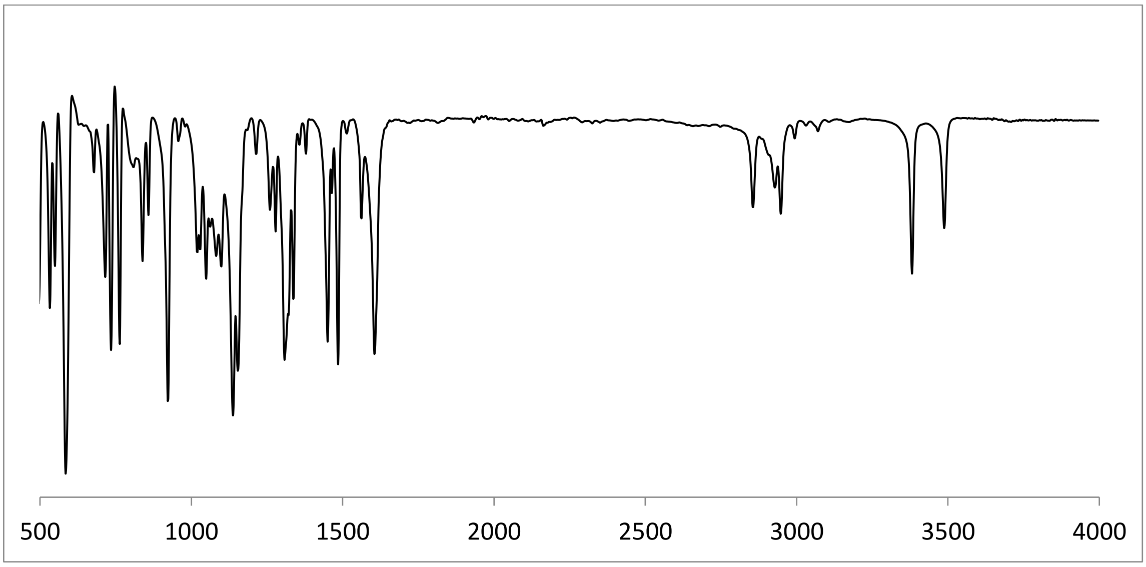

Figure S32: Infrared spectrum of *N*-Piperidinyl-*o*-sulphanilamide (**5**)

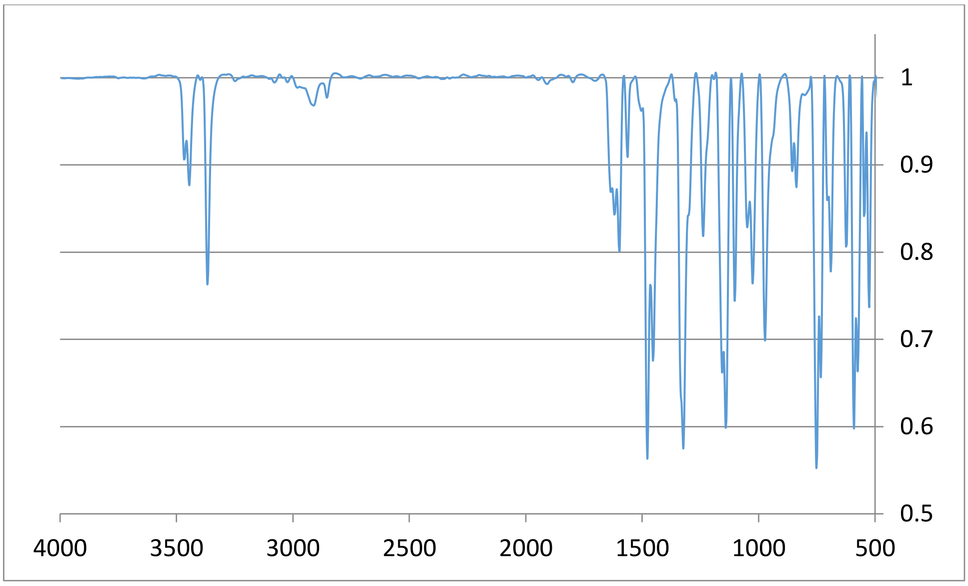

Figure S33: Infrared spectrum of *N*-Indolinyl-*o*-sulphanilamide (**6**)

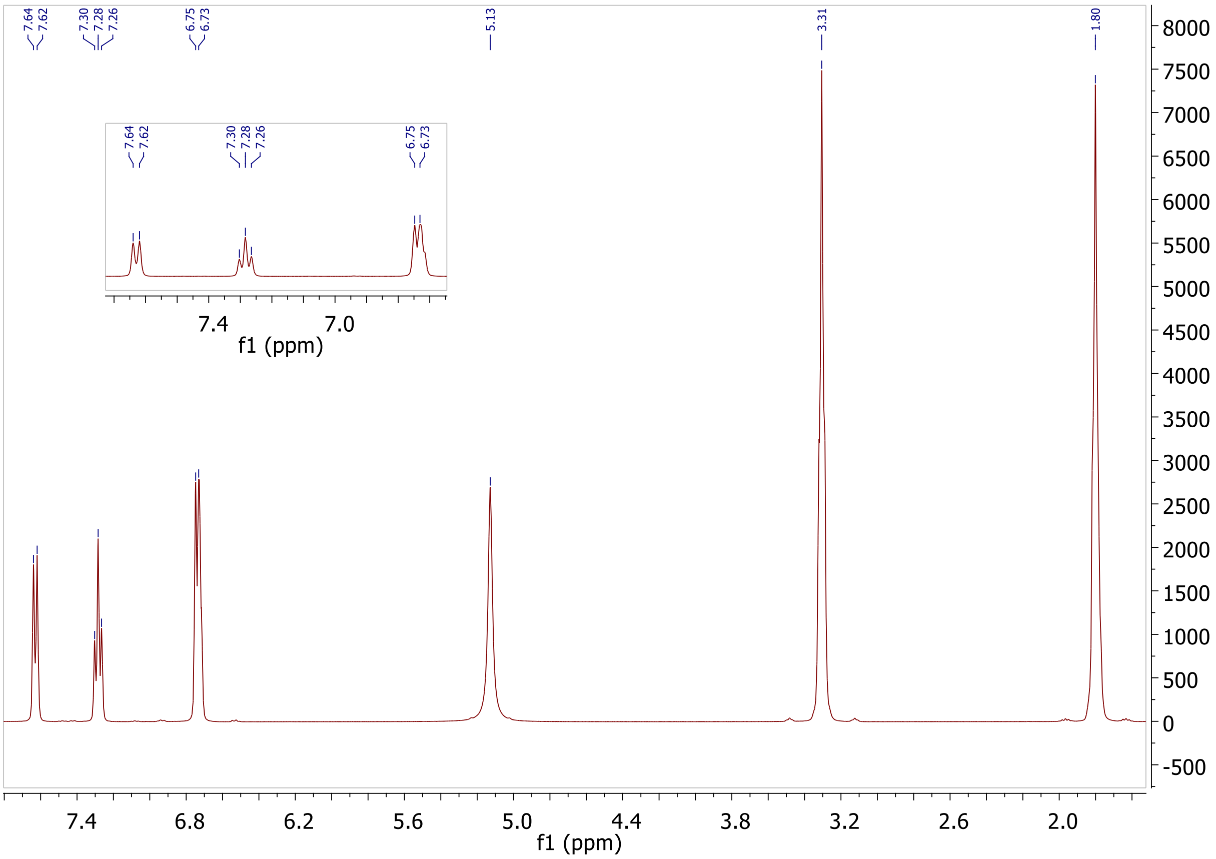

Figure S34: ¹H-NMR spectrum of *N*-Pyrrolidinyl-*o*-sulphanilamide (**4**)

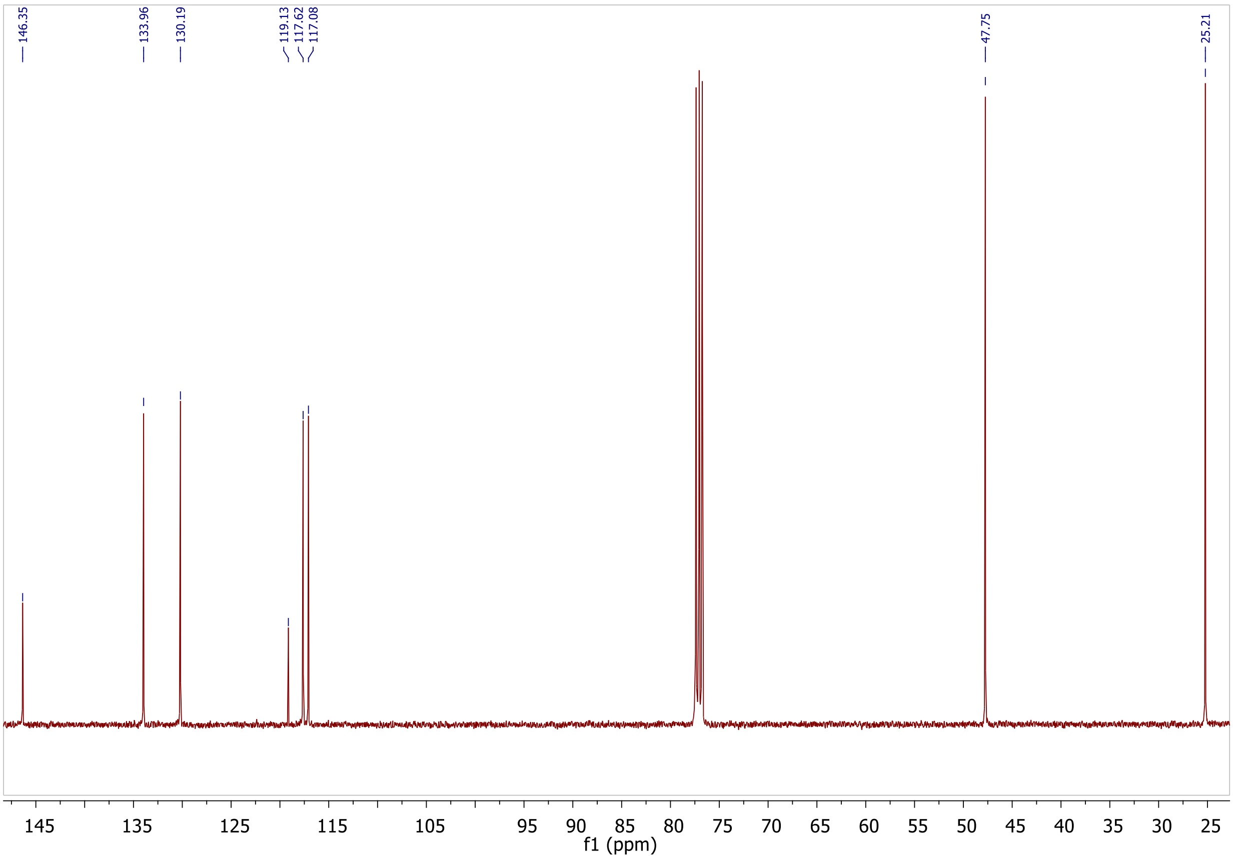

Figure S35: ^{13}C-NMR spectrum of *N*-Pyrrolidinyl-*o*-sulphanilamide (**4**)

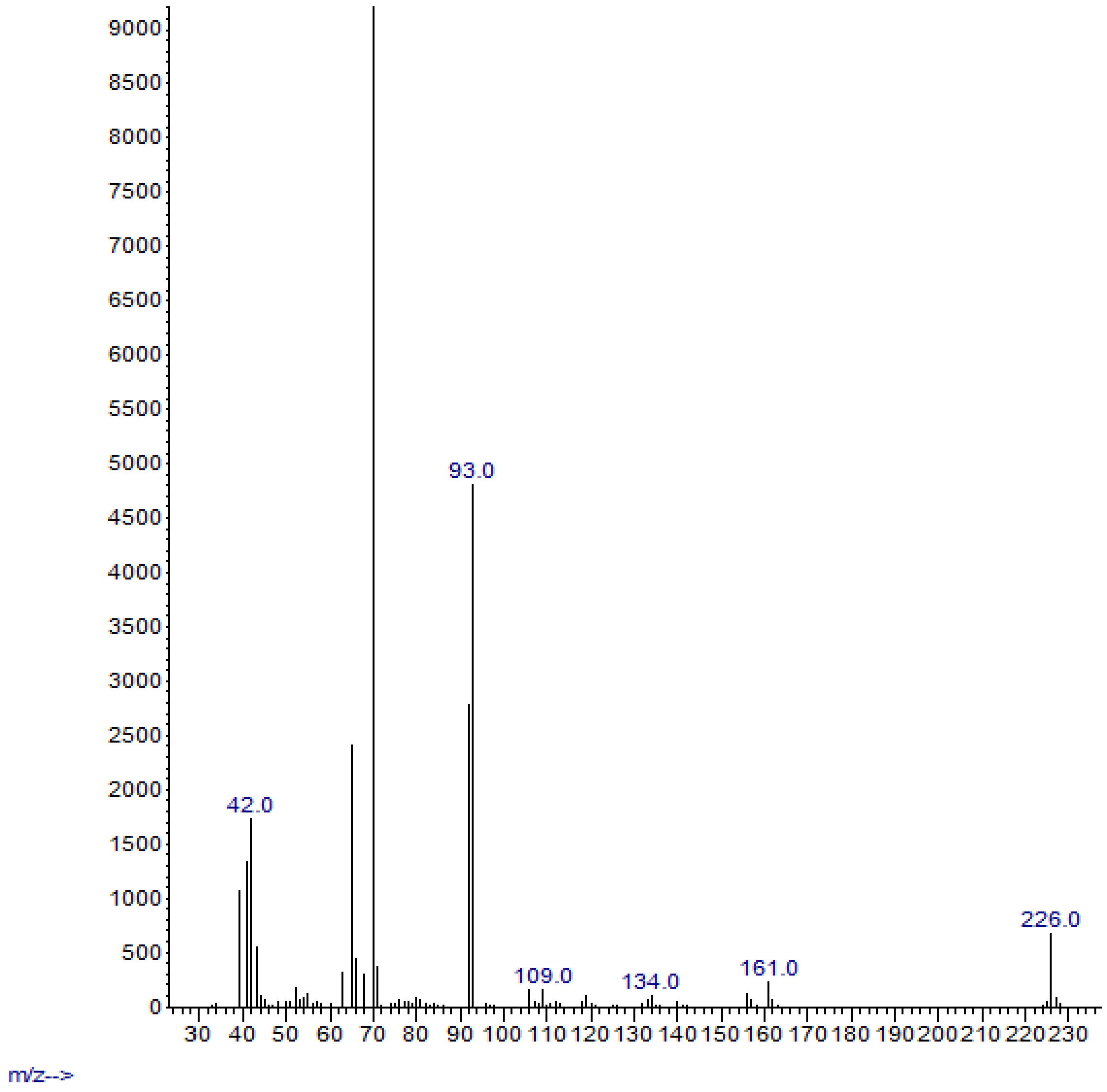

Figure S36: MS spectrum of *N*-Pyrrolidinyl-*o*-sulphanilamide (**4**)

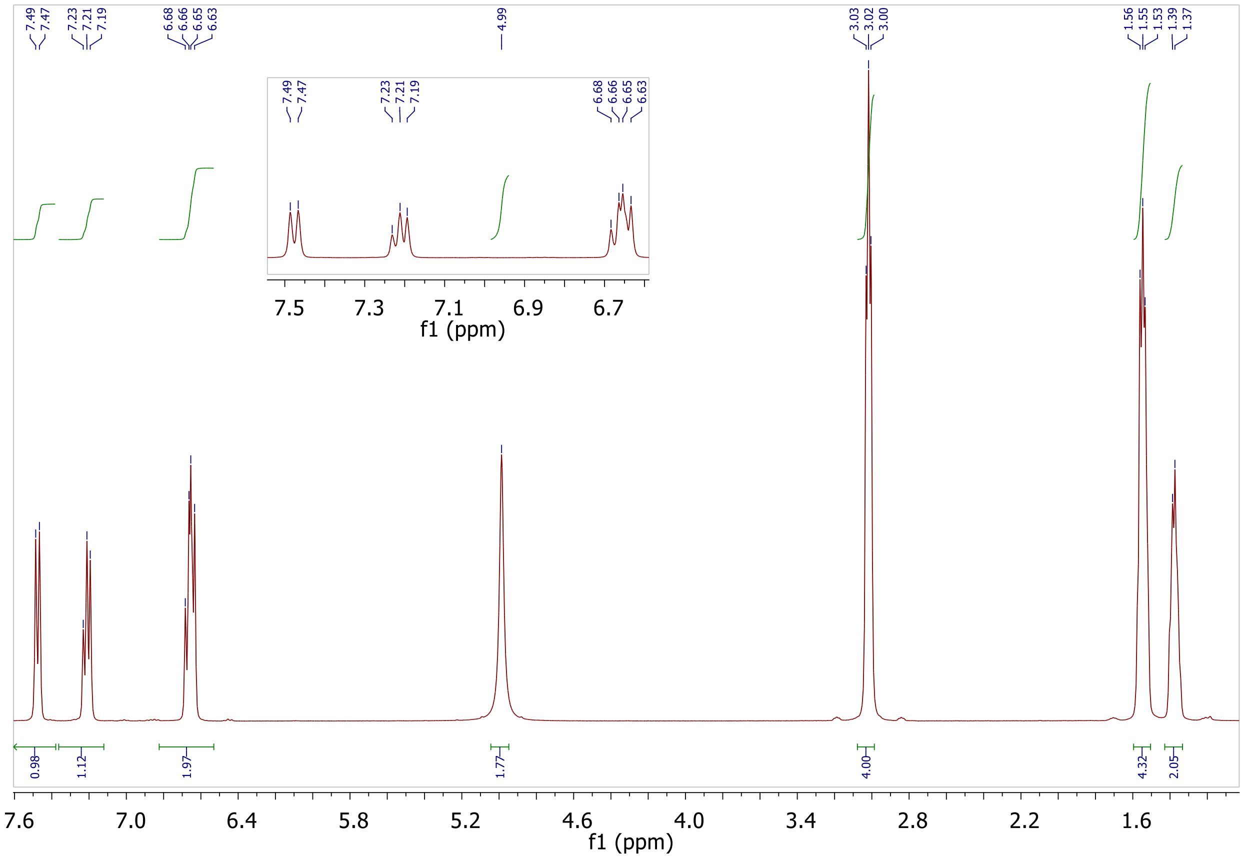

Figure S37: ^{1}H-NMR spectrum of *N*-Piperidinyl-*o*-sulphanilamide (**5**)

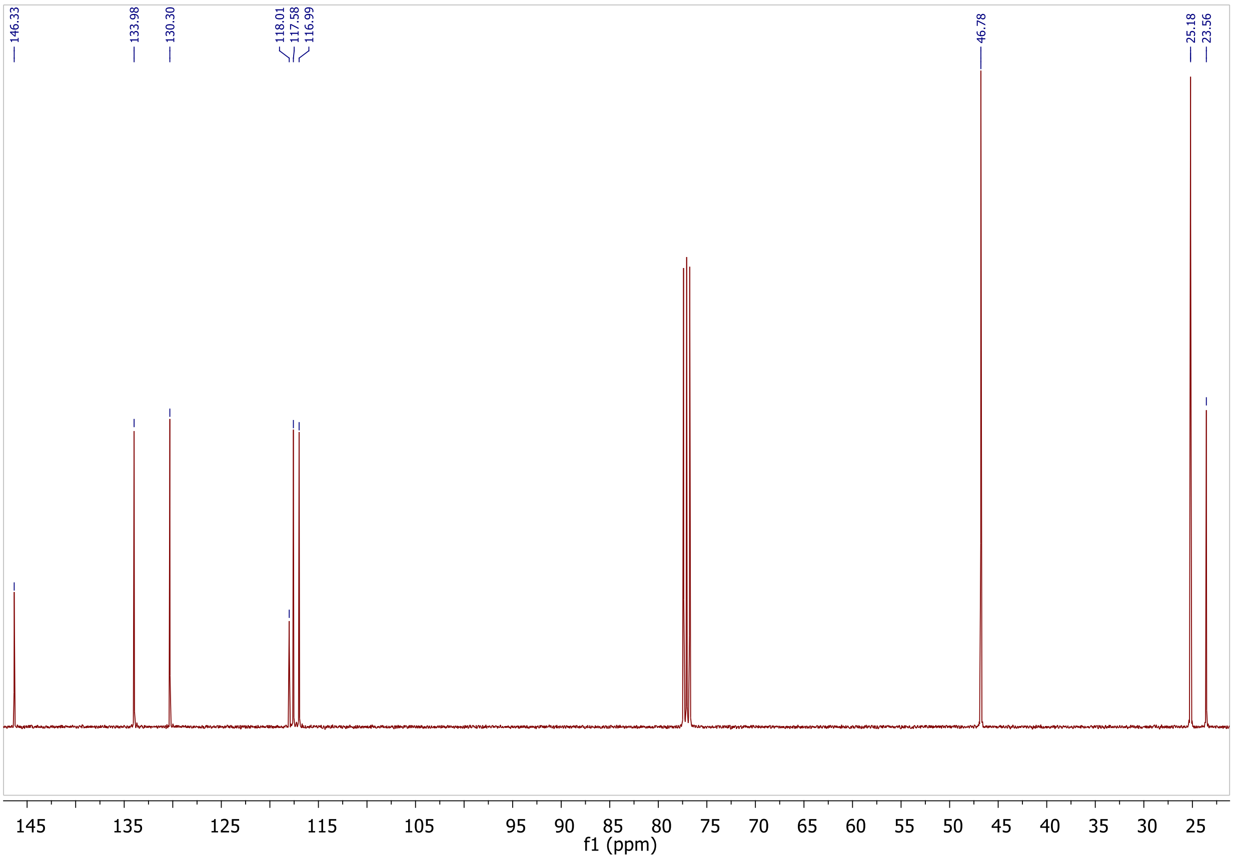

Figure S38: ^{13}C-NMR spectrum of *N*-Piperidinyl-*o*-sulphanilamide (**5**)

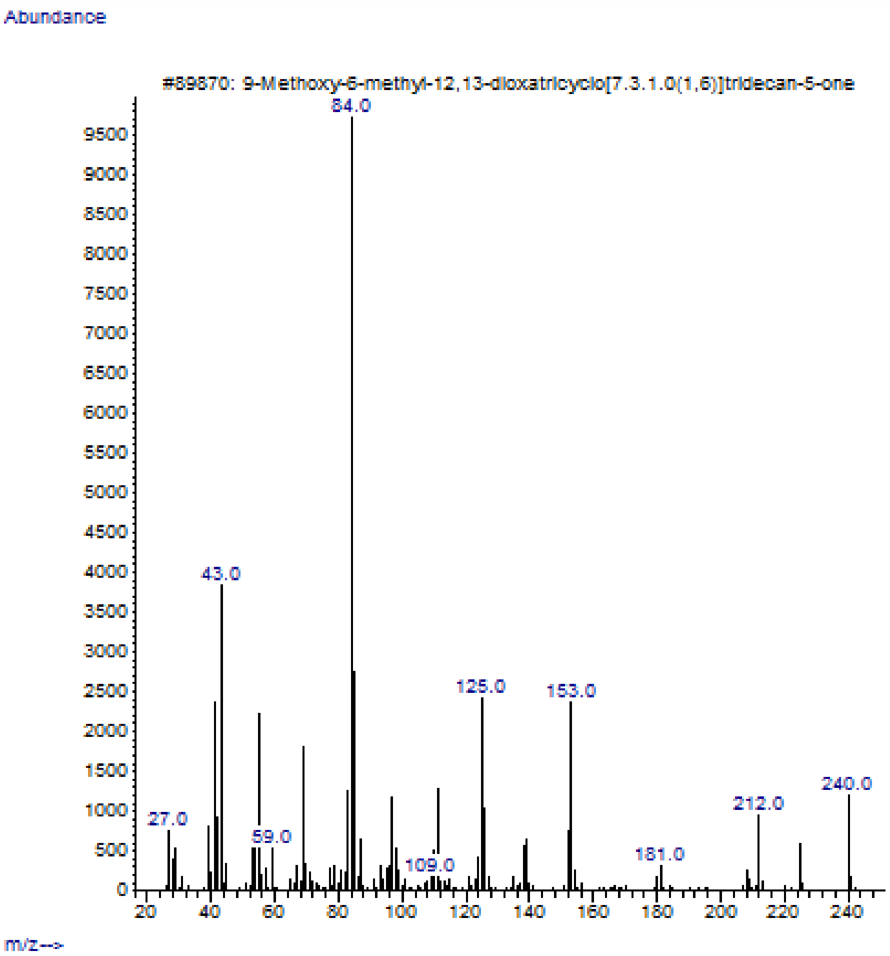

Figure S39: MS spectrum of *N*-Piperidinyl-*o*-sulphanilamide (**5**)

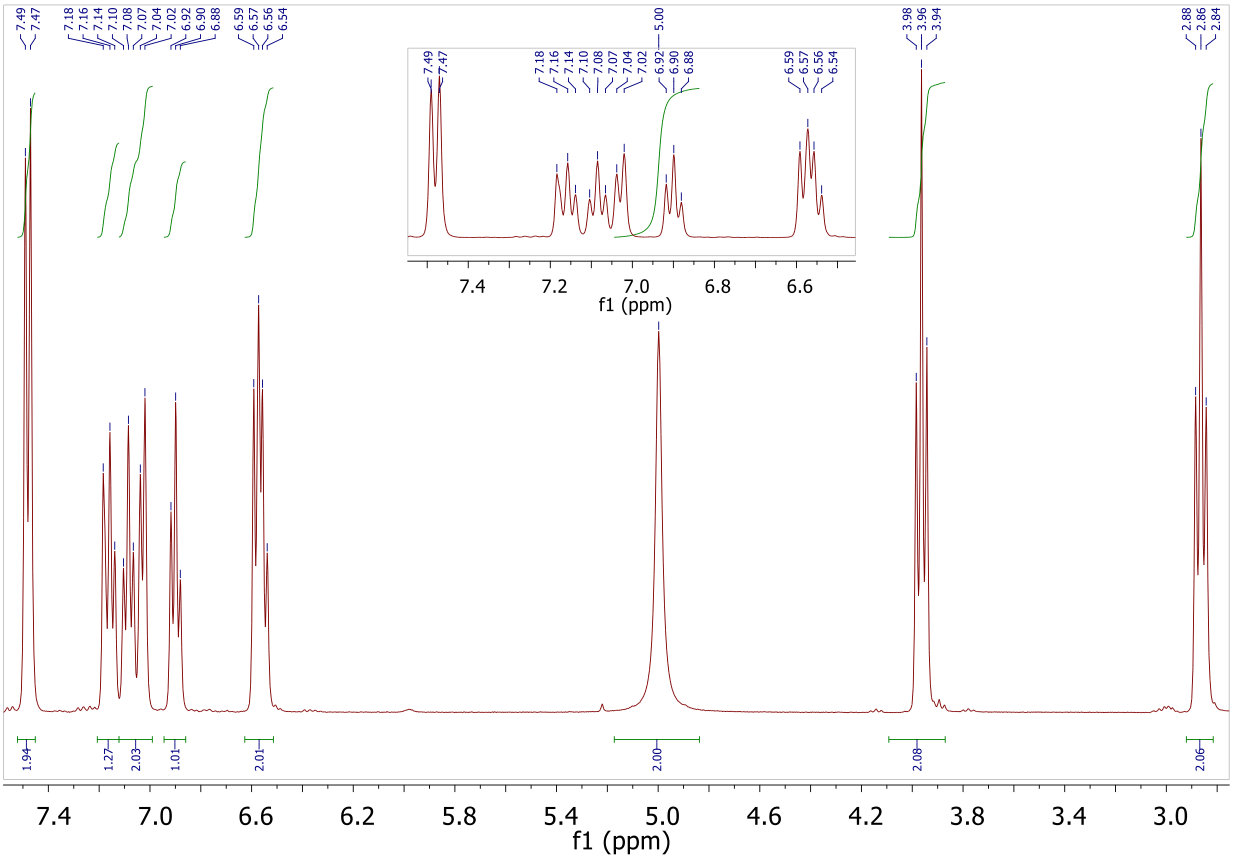

Figure S40: ^{1}H-NMR spectrum of *N*-Indolinyl-*o*-sulphanilamide (**6**)

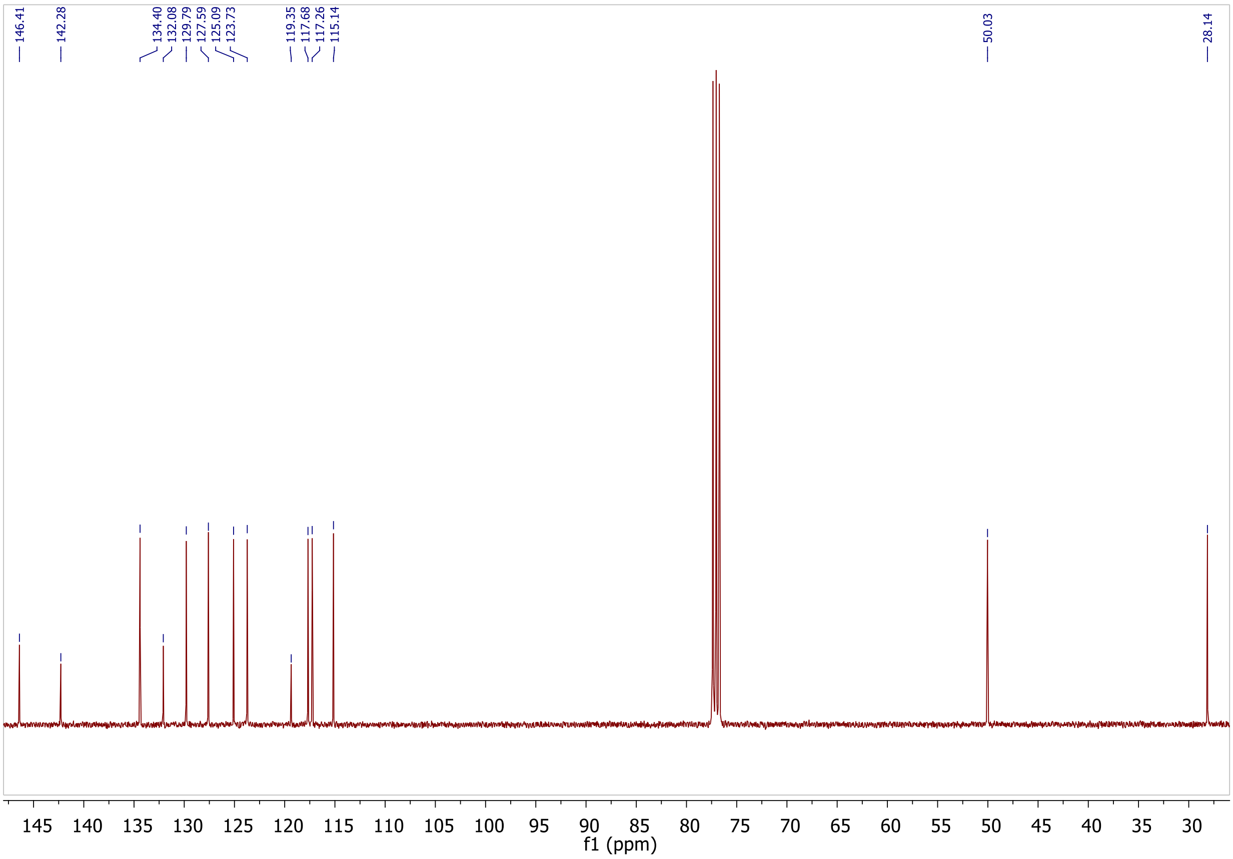

Figure S41: ^{13}C-NMR spectrum of *N*-Indolinyl-*o*-sulphanilamide (**6**)

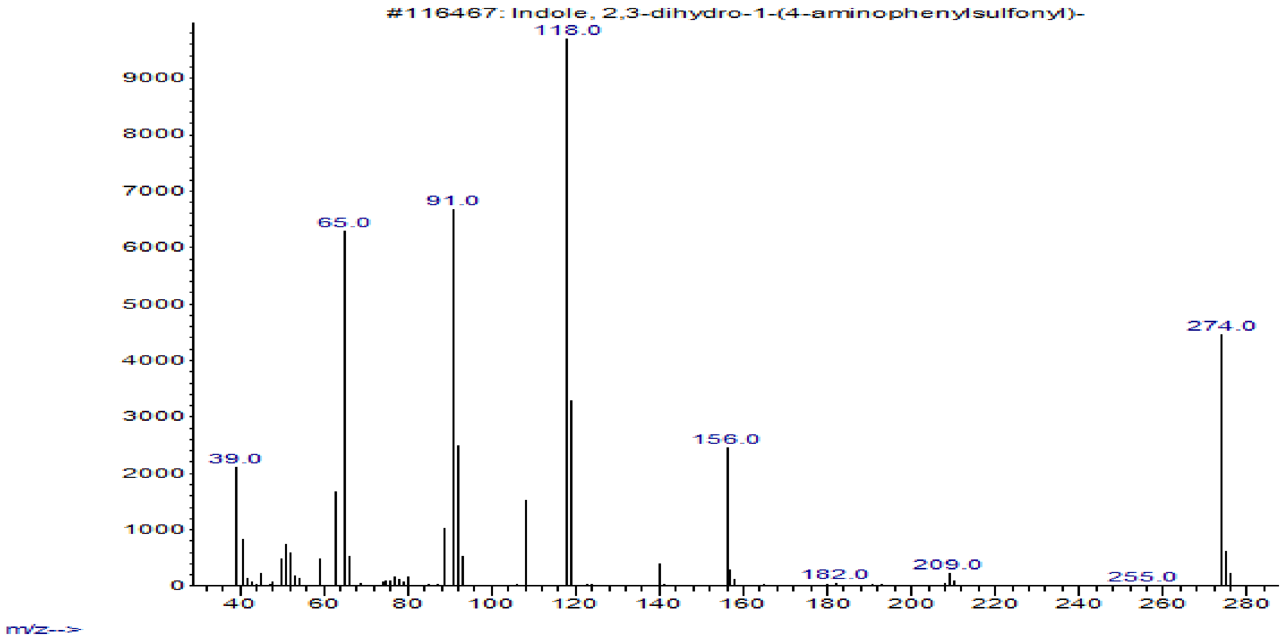

Figure S42: MS spectrum of *N*-Indolinyl-*o*-sulphanilamide (**6**)
